# The biological basis of degenerative disc disease: proteomic and biomechanical analysis of the canine intervertebral disc

**DOI:** 10.1186/s13075-015-0733-z

**Published:** 2015-09-05

**Authors:** William Mark Erwin, Leroi DeSouza, Martha Funabashi, Greg Kawchuk, Muhammad Zia Karim, Sarah Kim, Stefanie Mӓdler, Ajay Matta, Xiaomei Wang, K. Arne Mehrkens

**Affiliations:** Toronto Western Research Institute, Toronto Western Hospital, Toronto, ON M5T 2S8 Canada; Divisions of Neurological and Orthopaedic Surgery, University of Toronto, Toronto, ON Canada; Canadian Memorial Chiropractic College, North York, ON Canada; Department of Chemistry, York University, Toronto, ON Canada; Department of Physical Therapy, University of Alberta, 8205 114 Street, 2-50 Corbett Hall, Edmonton, AB T6G 2G4 Canada; Spinal Surgery, University of Basel, Basel, Switzerland

## Abstract

**Introduction:**

In the present study, we sought to quantify and contrast the secretome and biomechanical properties of the non-chondrodystrophic (NCD) and chondrodystrophic (CD) canine intervertebral disc (IVD) nucleus pulposus (NP).

**Methods:**

We used iTRAQ proteomic methods to quantify the secretome of both CD and NCD NP. Differential levels of proteins detected were further verified using immunohistochemistry, Western blotting, and proteoglycan extraction in order to evaluate the integrity of the small leucine-rich proteoglycans (SLRPs) decorin and biglycan. Additionally, we used robotic biomechanical testing to evaluate the biomechanical properties of spinal motion segments from both CD and NCD canines.

**Results:**

We detected differential levels of decorin, biglycan, and fibronectin, as well as of other important extracellular matrix (ECM)-related proteins, such as fibromodulin and HAPLN1 in the IVD NP obtained from CD canines compared with NCD canines. The core proteins of the vital SLRPs decorin and biglycan were fragmented in CD NP but were intact in the NP of the NCD animals. CD and NCD vertebral motion segments demonstrated significant differences, with the CD segments having less stiffness and a more varied range of motion.

**Conclusions:**

The CD NP recapitulates key elements of human degenerative disc disease. Our data suggest that at least some of the compromised biomechanical properties of the degenerative disc arise from fibrocartilaginous metaplasia of the NP secondary to fragmentation of SLRP core proteins and associated degenerative changes affecting the ECM. This study demonstrates that the degenerative changes that naturally occur within the CD NP make this animal a valuable animal model with which to study IVD degeneration and potential biological therapeutics.

**Electronic supplementary material:**

The online version of this article (doi:10.1186/s13075-015-0733-z) contains supplementary material, which is available to authorized users.

## Introduction

Degenerative disc disease (DDD) affects the cellular and extracellular compartments of the entire intervertebral disc (IVD), often leading to associated pain and disability. The specific compartments of the disc affected by degenerative changes include the inner nucleus pulposus (NP), the annulus fibrosus (AF), the transition zone interposed between the NP and AF, and the vertebral end plates (VEPs). All of these components, in addition to vital extracellular matrix (ECM) molecules within the IVD NP, such as proteoglycans, contribute to the function of the IVD as a hydrophilic, multiaxial, viscoelastic, and flexible joint between vertebrae [1–3]. Within the IVD NP ECM, the small leucine-rich proteoglycans (SLRPs) are involved with fibrillogenesis and interact and bind growth factors dependent upon the integrity of their core proteins and functioning glycosaminoglycan (GAG) side chains [4].

The non-degenerative NP, with its high water content, has biomechanical characteristics that are more fluidlike than those of the more fibrous, degenerative NP (with inferior hydrophilic properties), which behaves more like a solid [5]. However, with increasing degeneration, there is fragmentation of the SLRP core proteins, leading to a loss of ECM homeostasis, water content, and further biochemical and biomechanical compromise [6]. With progressive degeneration, although the overall collagen content remains relatively stable, there is a shift in the expression of collagen type II to collagen type I within the nucleus [4, 7–9]. This shift in the secretion of collagen, along with degradation of other ECM proteins within the NP, may contribute to aberrant biomechanical properties, pain, reflexive muscular spasm, and disability.

In an effort to mimic human DDD, non-physiologic perturbations intended to create IVD degeneration, such as needle puncture, have been developed in animals such as the rat, mouse, rabbit, pig, sheep, and canine. However, there are tremendous differences in the morphologies and cellular phenotypes of the IVDs of many animal models that bear varying resemblance to the human condition. However, two subspecies of canine differentially develop DDD in the absence of any external stimulus. Specifically, the chondrodystrophic (CD) canine (e.g., beagle or dachshund) develops DDD early and profoundly by the age of 1 year [10]. This is in stark contrast to the non-chondrodystrophic (NCD) animal, which does not develop DDD until much later in life, if at all [10–13]. Zhou et al. proposed that the histological, radiological, and biochemical changes within the CD animal resemble the pathology of human DDD [14, 15]. Notably, DDD is diagnosed and treated both medically and surgically the same in dogs as in humans [14].

The gross pathological and biochemical characteristics (including matrix metalloproteinase [MMP] activity and GAG content) are similar in canine and human IVD NP, such that the hallmarks of DDD at different stages (both dogs and humans) include chondroid cell clusters, disorganization of the AF, and increasing appearance of clefts and cracks of increasing severity [14]. These histological characteristics are supported by magnetic resonance imaging (MRI) scans showing striking similarities between the appearance of DDD in the different stages of degeneration in humans and dogs [14]. However, despite these imaging comparisons and assessment of GAG content, most comparisons are qualitative in nature, and the degenerative phenotype often ascribed to the CD IVD NP has not been quantified. In the present study, we used high-throughput quantitative proteomic analysis to compare the secretome of the CD canine (beagle) IVD NP (similar to the human disc) with that of the NCD (mongrel) canine subspecies, which is analogous to the IVD of a healthy, young human. We validated our proteomic investigation using Western blot analysis and immunohistochemical assays for both NCD and CD canines. After proteoglycan extraction, we characterized the integrity of the core proteins of the SLRPs decorin and biglycan obtained from the IVD NP of these animals. Finally, we quantified and compared the biomechanical properties of spinal motion segments obtained from both subspecies using robotic biomechanical testing.

## Methods

### Sample collection of NCD and CD intervertebral disc nucleus pulposus

All procedures involving the sacrifice of the NCD and CD dogs and tissue handling were carried out in accordance with established animal use protocols at the University Health Network, University of Toronto, ON, Canada, and in compliance with the regulations of the Canadian Council on Animal Care. We removed the T5-L6 spinal motion segments immediately after the animals were sacrificed, and then we placed all the specimens on ice. All canines used for the study were between 20 and 24 months of age and equally divided by sex. For biomechanical studies, the L4-L5 motion segment was recovered in its entirety, cleared of all soft tissues including ligaments, labeled, and immediately stored at −80 °C. For proteomic and biochemical analyses, we performed a wide laminectomy on the remainder of the spine using a Stryker saw (Stryker, Kalamazoo, MI, USA) followed by clearing of all soft tissues. After the posterior elements were removed, the spine was washed with Clidox (Pharmacal Research Laboratories, Waterbury, CT, USA) and Betadine (Purdue Products, Stamford, CT, USA) and cooled on ice. Thereafter the discs were incised immediately adjacent to the inferior end plate with a number 10 -blade, and the NP was removed in its entirety as per our established methods [16, 17]. The NP was placed into warmed physiological phosphate-buffered saline (PBS; 0.1 M, pH 7.4), rinsed, and then divided for isobaric tags for relative and absolute quantitation (iTRAQ) or immunohistochemical analysis. We were meticulous in the selection of the IVD NP used for these experiments, such that all NCD discs used were of the jellylike, mucoid, translucent appearance and all CD discs appeared fibrocartilaginous and pulpy, consistent with the degenerative phenotype widely used to describe this animal. For histological preparation, the removed IVD NP was fixed in 10 % formalin, placed in appropriately labeled cassettes, and processed using 10 % neutral buffered formalin, ascending grades of ethanol, two changes of the clearing agent xylol, and infiltrated with three changes of molten paraffin (Paraplast) for 12 h. After processing, the samples were placed into an automated embedding unit, and one sample at a time was moved from its cassette into a metal mold, appropriately oriented, filled with molten paraffin, and cooled on a cold plate and then sent for analysis.

### Sample processing and iTRAQ labeling

The NP was obtained in its entirety from the IVD of CD (CD1–CD4; beagle) and NCD (NCD1–NCD4; mongrel) canines immediately after the dogs were killed as per our established methods [16]. The IVD NP thus obtained was washed three times in 1 ml of cold PBS (0.1 M, pH 7.2) and homogenized in 0.5 ml of PBS with a protease inhibitor cocktail (Sigma-Aldrich, St. Louis, MO, USA). Next, the mixture was centrifuged at 15,000×*g* for 20 minutes, and the supernatant was collected. The total protein concentration was determined using a Bradford-type colorimetric assay as described elsewhere [18]. For each set, clarified tissue lysates were individually denatured, alkylated, digested with trypsin, and labeled with iTRAQ labels (SCIEX, Concord, ON, Canada). The sets were grouped as follows:

- Set 1: iTRAQ label 114: mongrel, M1; iTRAQ label 115: mongrel, M2; iTRAQ label 116: M3; iTRAQ 117: reference sample
- Set 2: iTRAQ label 114: mongrel, M4; iTRAQ label 115: beagle, B1; iTRAQ label 116: reference sample; iTRAQ label 117: beagle, B2
- Set 3: iTRAQ label 114: beagle, B3; iTRAQ label 115: reference sample; iTRAQ label 116: beagle, B4

The reference sample consisted of equal amounts of total protein from each of the eight samples used in this study, thereby providing a global reference that was common to all three sets. We randomized the labeling of the reference sample in each set to prevent any inadvertent introduction of labeling bias. We then pooled the labeled samples to form three 4-plex iTRAQ sets.

### Mass spectrometry and data analysis

Each iTRAQ set was processed by two-dimensional liquid chromatography–tandem mass spectrometry analysis as described earlier [18]. Briefly, the samples were separated offline using strong cation exchange (SCX) chromatography and fractionated into 30 SCX fractions. Each of these SCX fractions was then dried and redissolved in a minimal volume (20–30 μl) of 0.1 % formic acid. The redissolved fractions were injected into a reversed-phase (RP) trap column, where they were desalted before being eluted into an analytical nano liquid chromatography (LC) RP column (75-μm internal diameter (i.d.) × 150 mm). Elution and separation on the nano LC column were effected using a binary gradient of water and acetonitrile with 0.1 % formic acid at a 200 nl/min flow rate. Eluting peptides were analyzed on a 5600 Triple time-of-flight instrument (SCIEX) in information-dependent acquisition mode. Each set was analyzed a minimum of two times, and the resulting MS data were then analyzed using the ProteinPilot software (v4.0) package from SCIEX using a mammalian database. A false discovery rate (FDR) was calculated to provide a measure of confidence in the proteins reported. To minimize redundancy between proteins reported in the three individual iTRAQ sets and to ensure consistency of reported isoforms from one set to the next, the results of the three sets were aligned using a Microsoft Excel-based (Microsoft, Redmond, WA, USA) protein alignment template as described elsewhere [19]. We first generated a master list of all the proteins identified in this study by performing a search of the combined data from all three iTRAQ sets and duplicate runs using ProteinPilot.

Relative quantification of proteins, which was simultaneously performed using ProteinPilot, was based on the areas of the iTRAQ signature ion peaks. Only peptides not shared with other reported proteins (or group) contribute to the overall ratios reported for the protein. The overall protein ratios reported represent a weighted average of the ratios of the contributing unique peptides, where the weighting factor is determined by the percentage error of the individual peptide ratios. Further, the reported ratios were normalized using a factor, termed the *applied bias*, that is calculated based on the assumption that the majority of the proteins being compared between the samples in a set are expressed at similar levels.

### Western blot analysis

As further validation of the iTRAQ data, we chose to probe the same NP homogenate supernatants from which we generated samples for iTRAQ for the level of proteins secreted, as determined in our proteomic experiments. Equal amounts of tissue homogenates from one representative sample each of NCD and CD NP that was used for iTRAQ analysis and immunohistochemistry were subjected to Western blot analysis and probed for the levels of secreted fibromodulin, biglycan, and hyaluronan and proteoglycan link protein 1 (HAPLN1) as additional verification of our iTRAQ results. Briefly, 50 μg of total protein obtained from IVDs were resolved on SDS-PAGE gels. Proteins were then electrotransferred onto polyvinylidene difluoride membranes (Bio-Rad Laboratories, Hercules, CA, USA). After blocking with 5 % non-fat powdered milk in Tris-buffered saline (TBS; 0.1 M, pH 7.4), blots were incubated with either rabbit polyclonal antibody against fibromodulin (1:1000, catalog number sc-33772; Santa Cruz Biotechnology, Santa Cruz, CA, USA), goat polyclonal antibody against biglycan (1:1000, catalog number sc-27936; Santa Cruz Biotechnology), and goat polyclonal antibody against HAPLN1 (1:1000, catalog number sc-46826; Santa Cruz Biotechnology) at 4 °C overnight. We used protein lysates obtained from canine articular cartilage as positive controls. Membranes were incubated with the corresponding secondary antibody, horseradish peroxidase (HRP)-conjugated rabbit/goat anti-immunoglobulin G (anti-IgG; Bio-Rad Laboratories) diluted 1:10,000 in 1 % bovine serum albumin (BSA) for 2 h at room temperature. After each step, blots were washed three times with TBS with Tween 20 (0.1 %). Protein bands were detected by the enhanced chemiluminescence method (GE Healthcare Bio-Sciences, Pittsburgh, PA, USA) on Kodak Hyperfilm (Amersham; GE Healthcare, Little Chalfont, UK).

### Histology and immunohistochemistry

To validate the cellular morphology and characteristics of the discs obtained from both NCD and CD animals, we stained representative sections of the discs using hematoxylin and eosin and Safranin O. We performed immunohistochemical analysis for fibromodulin, HAPLN1, biglycan, decorin, and aggrecan using independent sets of paraffin-embedded sections of IVD NP from the same animals used for iTRAQ analysis. In brief, the sections were deparaffinized in xylene, hydrated in gradient alcohol, and pretreated in a microwave oven for 10 minutes at maximum power in Tris–ethylenediaminetetraacetic acid (EDTA) buffer (0.01 M, pH 9.0, 0.05 % Tween-20) for antigen retrieval. The sections were incubated with hydrogen peroxide (0.3 % vol/vol) in PBS (0.1 M, pH 7.2) for 15 minutes to quench the endogenous peroxidase activity, followed by blocking with 5 % BSA to preclude non-specific binding. Thereafter the slides were incubated with fibromodulin, HAPLN1, biglycan, decorin, and aggrecan antibodies for 16 h at 4 °C. The primary antibody was detected using the streptavidin–biotin complex with the Dako LSAB+ kit (Dako Cytomation, Glostrup, Denmark) and diaminobenzidine as the chromogen. All procedures were carried out at room temperature unless otherwise specified. The slides were washed three times after every step using PBS containing 0.025 % Triton X-100, and finally the sections were counterstained with Mayer’s hematoxylin and mounted with DPX mountant (distrene, plasticizer, and xylene). We replaced the primary antibody with isotype-specific non-immune goat/rabbit IgG for negative controls, and all sections were evaluated by light microscopic examination.

### Proteoglycan extraction and SLRP assay

As described above, entire NPs were removed from five spinal segments of each of three CD and NCD donor animals, all of which had the same gross morphological appearance and were within the same age range as the donors of our iTRAQ samples. Proteoglycans were then extracted using GuHCl according to the method described by Melrose et al. [6, 20]. NCD and CD NP were diced, weighed (approximately 0.4 g wet weight), and extracted with 10 volumes of 4 M GuHCl 100 mM acetate buffer (pH 5.8 containing 20 mM EDTA, 100 mM 6-amino hexanoic acid, 25 mM benzamidine) for 40 h at 4 °C with constant end-over-end mixing. The tissue residue was removed by centrifugation, and the supernatant was concentrated by ultrafiltration (Amicon Ultra-15, 15 ml, 3000 kDa, catalog number UFC900308; EMD Millipore, Billerica, MA, USA), dialyzed against three changes of deionized cold water using a D-Tube Dialyzer Maxi (MWXO 3.5 kDa, catalog number 71508-3; EMD Millipore) with a fourth overnight dialysis. The dialyzed extracts were then freeze-dried. The freeze-dried proteoglycans thus extracted were dissolved in 2.1 ml of digestion buffer (0.1 M Tris, 0.03 M acetate, pH 6.5) overnight at 4 °C with constant shaking, and the protein content of the dissolved extracts was calculated using a Bradford assay. Briefly, 500 μg of protein from NCD and CD samples were digested with 1.0 U of chondroitinase ABC (C3667; Sigma-Aldrich) and 0.05 U of keratinase (G6920; Sigma-Aldrich) overnight at 37 °C. Next, 30 μg of total protein obtained from the extracted proteoglycans (digested and undigested) were mixed with SDS loading buffer and dithiothreitol, electrophoresed, and visualized by Western blotting as described above. For the decorin and biglycan Western blot primary antibodies, we used a rabbit polyclonal antibody against decorin (1:200, catalog number NBP1-57923; Novus Biologicals, Littleton, CO, USA) and a goat polyclonal antibody against biglycan (1:200, catalog number sc-27936; Santa Cruz Biotechnology). The secondary antibodies were both HRP conjugates. We used a goat anti-rabbit IgG (heavy and light chains, catalog number (170-6515; Bio-Rad Laboratories) and a rabbit anti-goat IgG (heavy and light chains, catalog number#A24452; Life Technologies, Carlsbad, CA, USA), both used at 1:10,000 dilution.

### Biomechanical testing

**Fig. 1:**
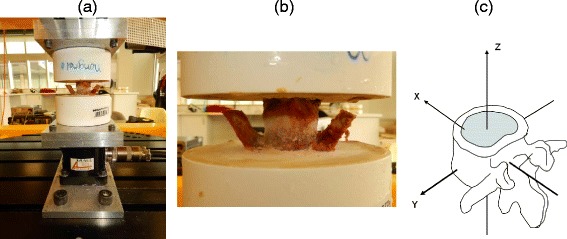
Robotic biomechanical setup for intervertebral disc (IVD) motion segments. **a** and **b** Potted IVD motion segment mounted upon the robotic platform. **c** The rotational axis x, y and z reflect the three dimensions of movement afforded by biomechanical testing

We harvested the L4-L5 lumbar motion segments from CD and NCD animals within 1–4 h after they were sacrificed. These segments were removed from the spines from which the IVDs were subjected to both iTRAQ analysis and Western blot analysis. CD and NCD canines (*n*=6 samples each) were age-matched (20–24 months) and had average weights of 10 kg and 14 kg, respectively. Following lumbar segment harvesting, the muscle tissues, intertransverse ligaments, and supra- and interspinous ligaments were carefully dissected, leaving the posterior joints and the IVD intact. All segments were kept frozen at −20 °C until testing. The IVD dimensions of each specimen were measured, and the cross-sectional areas were calculated. Before testing, the lumbar segments were thawed at room temperature, and pilot holes were then drilled into each VEP. Each hole accommodated two anchor screws (#3 × 25.3 mm) in the superior L4 end plate and one screw (#8 × 43 mm) in the inferior L5 end plate, perpendicular to the middisc plane. Dental stone (Modern Materials; Heraeus Kulzer, South Bend, IN, USA) was prepared according to the manufacturer’s recommendations and used to cement the segments within 3.5-inch–diameter plastic pots. The two vertebra composing the motion segments (inclusive of the intervening IVD) were seated within opposing pots using a standardized template, and cement was added to the pot until two-thirds of the vertebral body height was covered, leaving the IVD exposed (Fig. 1). Each segment was kept hydrated with gauze soaked in isotonic saline and covered in plastic wrap during tissue preparation, potting, and testing. The pot containing the caudal vertebra (L5) was fixed to a stationary crossbeam, and the pot containing the cephalad vertebra (L4) was fixed to a six-axis load cell (AMTI MC3A-100, Advanced Mechanical Technology, Watertown, MA, USA), which in turn was attached rigidly to the parallel robot (Parallel Robotic Systems, Hampton, NH, USA). This robot is composed of a rigid platform suspended by six rigid struts of fixed length. Each strut was attached to an electromechanical motor that travels about a circular track to alter the position and/or orientation of the robot platform and thus the potted motion segment. Custom computer software (National Instruments, Austin, TX, USA) was employed to control robot position and orientation. The resolution of the robot is a function of the motor performance (0.05 mm with a repeatability of 0.025 mm; Mikrolar, Hampton, NH, USA), which translates into a linear resolution of less than 1 μm and an angular resolution of approximately 0.001 degree [20].

The methodology used for biomechanical testing involved a standardized procedure according to an established testing protocol [21, 22]. Specimens were tested using an unconstrained force control system that permitted the application of a desired load in a specific axis of motion without any constraints on the segment’s motion behavior, regardless of the specimen condition (e.g., intact, injured) [23]. Specifically, we employed our software to position the specimen in a neutral pose (e.g., loads and moments in all directions were minimized), after which a bending moment about all three axes of movement (i.e., flexion and extension, lateral bending, and axial rotation) (Fig. 1c) was applied until the moment obtained from the load cell reached a maximum predetermined value. When the maximum moment was reached in the positive direction, the direction of rotation was reversed to provide a corresponding negative moment. Based on preliminary tests conducted in three additional CD specimens (not included in this study), maximum moments of newton meters were predetermined for flexion and extension, ±3 Nm for lateral bending, and ±4.5 Nm for axial rotation [24, 25]. The robot’s displacement during specimen rotation was recorded directly through a software query via the robot. This query and collection of specimen moment and load data occurred at 200 Hz. Five moment cycles around each Cartesian axis were performed at 0.1/s. The first two cycles were applied to precondition the specimens and load deformation data from the last three cycles used in the statistical analysis [26, 27]. The specimens’ moment and rotation data were plotted, and specific point displacements were obtained at the neutral position, the midpoint of the ascending moment, the peak moment, and the midpoint of the descending moment for both positive and negative rotations.

### Statistical analysis

Before statistical analysis, the biomechanical data were normalized to the cross-sectional area of the IVDs to account for any differences in the sizes of the respective IVDs. We used the Shapiro–Wilk normality test to verify the normal distribution of data obtained from biomechanical testing, and numeric data were represented as the mean ± standard deviation. We compared normalized displacement mean values between the CD and NCD groups using an independent *t* test, with *p* < 0.05 considered statistically significant. The proteomic data are presented as weighted averages of the ratios of the relevant peptides (ratio calculated with respect to the reference standard) as described, and the data were normalized using the applied bias with β-actin as a normalizing factor. All proteins and peptides were statistically analyzed within the iTRAQ analysis software platform (ProteinPilot) after having achieved a 95 % confidence interval and 1 % global FDR cutoff plus up- or downregulation with respect to the reference pool of at least 0.5 of greater than 2.0-fold. The proteins and peptides identified by iTRAQ and their expression ratios are therefore not directly comparable to each other.

## Results

### Identification of differentially secreted proteins in nucleus pulposus of NCD vs. CD canines

Using a 95 % confidence interval and a cutoff of 1 % global FDR, we identified 377 proteins in our comparative proteomic analysis of proteins secreted within the NP of the two respective canine species (please see Additional file 1 Table S1). We expressed the levels of all proteins in each of the individual samples as ratios relative to the level of secretion of the same protein in the reference sample. The use of a common reference sample in all three sets therefore permitted direct comparisons of protein expression ratios across the different sets. We selected proteins showing consistent differential secretion in either the NCD or CD cases that were greater than twofold relative to the reference pool for further investigation. Following these criteria, our data analysis revealed 13 upregulated protein levels in CD (beagles) with respect to the reference pool and compared with the expression ratio of these proteins in the NCD animals. Interestingly, we observed dramatic differences between the secretion of a number of proteins critical for the function of the ECM in the IVD and cartilage. These included decorin, fibronectin, cartilage oligomeric matrix protein, cartilage intermediate layer protein, HAPLN1, biglycan, isoform B of proteoglycan 4, fibromodulin, and aggrecan core protein (Table 1).

**Table 1:** iTRAQ analysis of differential nucleus pulposus homogenate protein expression in NCD and CD dogs

### Verification of differential expression of proteins in nucleus pulposus (CD vs. NCD animals)

**Fig. 2:**
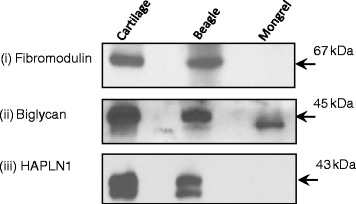
Western blots for fibromodulin, biglycan, and hyaluronan and proteoglycan link protein 1 (HAPLN1) expression in non-chondrodystrophic (NCD) and chondrodystrophic (CD) canine nucleus pulposus (NP) homogenates. *Lane (i)* depicts the expression of these proteins in articular cartilage used as a control. *Lanes (ii)* and *(iii)* represent the expression of these proteins in intervertebral disc NP homogenates for CD (beagle) and NCD (mongrel) samples. It is clear that there is strong expression in both articular cartilage and CD samples for all three proteins. However, in the NCD samples, fibromodulin and HAPLN1 are undetectable, and the expression of biglycan is markedly reduced

We determined the secretion of fibromodulin, HAPLN1, and biglycan in NCD and CD NP homenates using Western blot analysis with total protein extracted from articular cartilage as a control (see Methods section). We observed increased levels of fibromodulin, biglycan, and HAPLN1 in CD canines compared with NCD canines (Fig. 2). However, we were unable to determine the expression of β-actin or glyceraldehyde 3-phosphate dehydrogenase, used as loading controls in the Western blot experiments, because we used the same IVD NP homogenate samples for these Western blot experiments as we did for the iTRAQ experiments. These homogenates were not extracts of total protein but consisted of supernatants obtained from NP homogenates in PBS (as outlined above) and therefore contained insufficient β-actin such as would be obtained using a total protein lysate sample (as was done with the articular cartilage controls). We maintained strict controls over protein quantification (Bradford assays in quadruplicate) and scrupulously loaded the same total protein for each Western blot experiment. We repeated each experiment at least three times to confirm the protein expression in CD canines compared with NCD canines. The results of these analyses were consistent with the iTRAQ data.

### Histological and immunohistochemical analysis of intervertebral discs (CD vs. NCD canines)

**Fig. 3:**
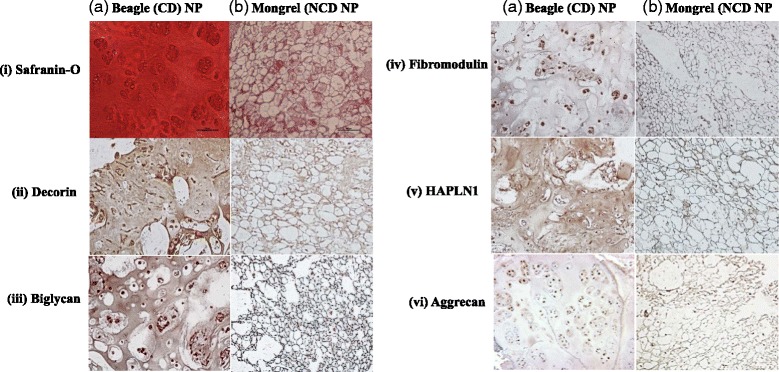
Comparative immunohistochemical analysis of non-chondrodystrophic (NCD) and chondrodystrophic (CD) canine nucleus pulposus (NP) expression and distribution of decorin, biglycan, fibromodulin, hyaluronan and proteoglycan link protein 1 (HAPLN1), and aggrecan. For all extracellular matrix (ECM) proteins, the NCD canine intervertebral disc NP reveals a cobweb appearance, demonstrating intense staining for all proteins located in the areas tightly between the cells. Immunostaining with decorin reveals diffuse intercellular staining with negative immunostaining within the large, physaliferous-appearing notochordal cells. This presentation is in contrast to the abundant staining of these proteins in every CD NP sample. Decorin, biglycan, and HAPLN1 reveal intense staining within the ECM, with abundant clusters of small numbers of cells present within the NP. Although the ECM staining is less intense than that for the other three proteins, fibromodulin and aggrecan immunostaining is present. Furthermore, the CD NP stained for aggrecan reveals intense pericellular immunostaining diffusely throughout the ECM that is much less cellular than the NCD NP staining. Safranin-O staining shows quite intense ECM staining in the CD NP, whereas the NCD sample demonstrates intense intercellular staining without large, acellular ECM areas rich in proteoglycan staining. The overall appearance of the CD NP bears a strong resemblance to a fibrocartilaginous phenotype that is distinctly different from the NCD canine NP

We used Safranin-O staining to evaluate proteoglycan content and distribution throughout the IVD NP and immunohistochemical methods to verify the differential expression of the proteins identified in our iTRAQ analysis (decorin, biglycan, fibromodulin, HAPLN1, and aggrecan) in IVDs obtained from beagles and mongrels (n=3 each). Representative images are shown in Fig. 3. All samples revealed the same overall morphology without exception. Safranin-O staining revealed a dramatic difference in appearance, with the CD NP demonstrating intense staining throughout the ECM and abundant clusters of chondrocyte-like cells. The NCD NP, however, revealed abundant physaliferous-appearing cells with positive Safranin-O staining at the intercellular spaces. The salient differences between the two canine subspecies was the abundant cell-free and intense Safranin-O staining within the ECM of the CD NP. The NCD NP contained a highly cellular NP with intense Safranin-O staining in the smaller intercellular space, and there were no chondrocyte-like clusters as seen in the CD IVD NP (Fig. 3). For all ECM proteins, the NCD canine IVD NP had a cobweb appearance with intense staining for all proteins located at the areas tightly between the cells. Immunostaining with decorin revealed diffuse intercellular staining. Much of the areas that were devoid of staining for any of the antibodies were contained within the large NP cells with a physaliferous appearance. This observation is in contrast to the abundant staining of these proteins in every CD NP sample. Decorin, biglycan, and HAPLN1 revealed intense staining within the ECM of the CD samples, with abundant clusters of small numbers of cells present within the NP (Fig. 3). Immunostaining for fibromodulin and aggrecan in the CD NP was less intense than for the other three proteins; however, the ECM staining was still more intense than that seen in the NCD NP (Fig. 3). Furthermore, the CD NP stained for aggrecan revealed intense pericellular immunostaining diffusely through the ECM that was much less cellular than the NCD NP. The overall appearance of the CD NP bore a strong resemblance to a fibrocartilaginous phenotype that was distinctly different from the NCD canine NP.

### SLRP expression in intervertebral disc nucleus pulposus

**Fig. 4:**
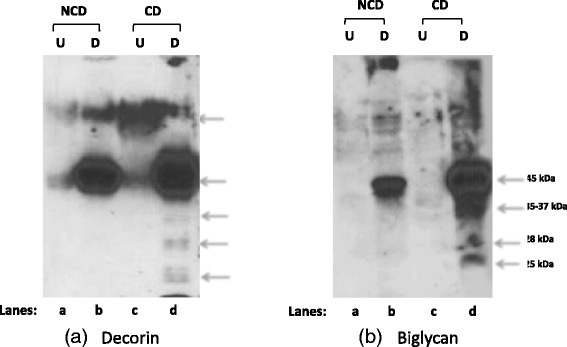
Western blots depicting the expression of decorin and biglycan after proteoglycan extraction from non-chondrodystrophic (NCD) and chondrodystrophic (CD) canine intervertebral disc (IVD) nucleus pulposus (NP). For each Western blot, *lane (a)* is undigested (U) proteoglycan extract from NCD (mongrel) and *lane (c)* is from CD (beagle) canine NP. *Lanes (b)* and *(d)* depict the detection of the specific small leucine-rich proteoglycan (SLRP) indicated above after digestion (D) with chondroitinase ABC and keratinase in NCD canine (*lane b*) and CD canine (*lane d*). **a** The decorin core protein is visualized appropriately at the 43 kDa molecular weight after digestion (*lane b*), with a clear, single band found from NCD NP extracts. NP extracts from CD canines (*lane d*) reveal fragmentation of decorin with multiple lower molecular weight bands visualized at 30, 25–20, and 17–15 kDa. **b** The biglycan core protein provides a clear, single band at the 45 kDa molecular weight in the NCD NP extract; however, the CD NP extract reveals multiple fragments of the core protein visualized at 37–35, 28, and 25 kDa

We hypothesized that the CD IVD NP represents a naturally occurring degenerative phenotype. Therefore, we extracted proteoglycans from NCD and CD canine NP to ascertain whether the core proteins in selected SLRP species were fragmented, as has been reported to occur in degenerative human discs [4, 20]. Interestingly, we observed multiple bands demonstrating fragmentation of the core proteins of decorin (30, 25, 20, 17, and 15 kDa) and biglycan (37, 28, and 25 kDa) in the CD samples, whereas the NCD samples were completely intact (Fig. 4). There was no evidence of non-specific binding of the antibodies in any samples, and these fragmentation bands visualized on the Western blots were entirely in keeping with what has been reported in human degenerative discs [4].

### Biomechanical robotic testing and analysis

**Fig. 5:**
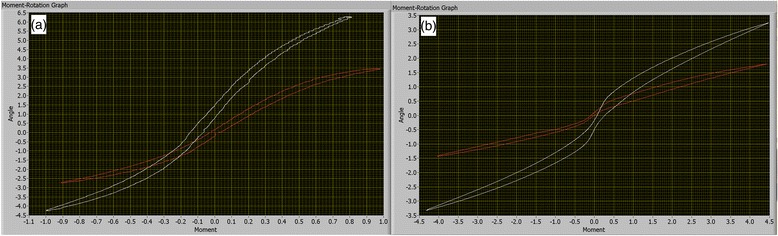
Averaged moment–rotation curves during (**a**) flexion and extension and (**b**) axial rotation movements for non-chondrodystrophic (NCD; *red*) and chondrodystrophic (CD; *white*) specimens. Differences observed between moment–rotation curves are indicative of greater stiffness during flexion and extension and axial rotation loading in the NCD motion segments compared with CD motion segments

The average IVD cross-sectional areas were 2.45 cm^2^ for the CD specimens and 3.10 cm^2^ for the NCD specimens. The averaged moment–rotation curve of CD and NCD specimens for flexion and extension and axial rotation movements are depicted in Fig. 5a and b, respectively. Differences observed between moment–rotation curves are indicative of greater stiffness during flexion and extension and axial rotational loading in the NCD motion segments compared with CD motion segments. Specifically, angular displacements in flexion and extension and axial rotation were significantly reduced (*p* < 0.05) in NCD specimens at the midpoint of both ascending and descending moments as well as at the peak moment (Table 2). There was no statistically significant difference between the neutral position displacements (*p* > 0.05).

**Table 2:** Analysis of angular displacements during the application of 1 Nm moment in flexion and extension and axial rotation motions

Movement		CD canines	NCD canines	*p* value	95 % CI
Flexion	Zero crossing displacement	0.01° (±0.02)	0.01° (±0.03)	0.850	−0.003, 0.024
	Halfway from zero to peak displacement	1.16° (±0.39)	0.73° (±0.16)	0.000	0.584, 0.876
	Peak displacement	2.29° (±0.79)	1.44° (±0.33)	0.000	1.150, 1.735
	Halfway from peak to zero displacement	1.15° (±0.39)	0.72° (±0.16)	0.000	0.573, 0.866
Extension	Zero crossing displacement	0.00° (±0.00)	0.00° (±0.00)	0.753	−0.001, 0.002
	Halfway from zero to peak displacement	−0.82° (±0.28)	−0.44° (±0.19)	0.000	−0.554, −0.220
	Peak displacement	−1.61° (±0.53)	−0.86° (±0.38)	0.000	−1.063, −0.434
	Halfway from peak to zero displacement	−0.78° (±0.27)	−0.43° (±0.20)	0.000	−0.514, −0.185
Right axial rotation	Zero crossing displacement	0.00° (±0.01)	0.00° (±0.03)	0.358	−0.002, 0.001
	Halfway from zero to peak displacement	0.55° (±0.25)	0.37° (±0.17)	0.016	0.036, 0.334
	Peak displacement	1.10° (±0.50)	0.73° (±0.35)	0.017	0.071, 0.663
	Halfway from peak to zero displacement	0.55° (±0.25)	0.36° (±0.17)	0.017	0.035, 0.331
Left axial rotation	Zero crossing displacement	0.00° (±0.01)	0.00° (±0.00)	0.492	−0.001, 0.001
	Halfway from zero to peak displacement	−0.60° (±0.20)	−0.38° (±0.24)	0.007	−0.374, −0.066
	Peak displacement	−1.20° (±0.41)	−0.73° (±0.48)	0.003	−0.778, −0.172
	Halfway from peak to zero displacement	−0.56° (±0.21)	−0.34° (±0.22)	0.005	−0.366, −0.069

*CD* chondrodystrophic, *CI* confidence interval, *NCD* Non-chondrodystrophic

Statistically significant differences between the NCD and CD motion segments in both flexion and extension and axial rotation motions are observed, indicating that the CD motion segments incur greater displacement (decreased stiffness) than the NCD motion segments

*p* < 0.05 was considered significant

## Discussion

In the present study, we used quantitative proteomic analysis, immunohistochemistry, Western blot analysis, and robotic biomechanical analysis to demonstrate salient molecular and biomechanical differences between the notochordal cell-rich (NCD) and notochordal cell-deficient (CD) IVD NP. In particular, supernatants developed from mechanically disrupted CD NP yielded far greater amounts of ECM products than NCD NP samples did. Furthermore, consistent with prior reports concerning degenerative human discs, we detected fragmentation of the core proteins of the vital SLRPs decorin and biglycan. However, within the NP of the NCD animal (just as is the case with healthy IVD NP), the core proteins of these same SLRPs were intact and we did not detect any non-specific binding in any samples. We also demonstrate that the notochordal cell-rich NCD IVD is stiffer and less variable than the CD IVD NP, which has reduced stiffness and more variable motion characteristics.

The SLRP family of proteins, including decorin, biglycan, and fibromodulin, is of considerable importance in the function and homeostatic regulation of the NP as well as articular cartilage. SLRPs facilitate interaction with fibrillar collagens, regulate fibrillogenesis and sequester growth factors such as epithelial growth factor and transforming growth factor (TGF)-β, in addition to their interaction with the cytokine tumor necrosis factor- α [20]. Intact SLRP GAG side chains interact with ECM proteins and facilitate fibril–fibril interaction, cellular proliferation, matrix adhesion, the modulation of cell growth, and the protection of the GAG side chains from proteolysis [4]. In this regard, decorin in particular performs a vital function with respect to its interaction with TGF-β, where decorin-TGF-β binding serves to provide a tissue reservoir of this ubiquitous growth factor that is essential for ECM homeostasis [28]. Decorin is reported to regulate collagen fibrillogenesis and matrix maintenance via interaction with fibronectin and thrombospondin, and its expression increases in cartilaginous tissues with increased degeneration [28]. TGF-β is released into the ECM upon cleavage and fragmentation of the decorin core protein, and unbound TGF-β in turn leads to increased MMP-13 expression, downstream inflammation, and accelerated degeneration of the ECM that are inextricably linked with degenerative disease [6, 20].

Capello et al. [29] reported that NP cells obtained from CD and NCD canines assemble and process proteoglycans (aggrecan in particular) very differently, in large part owing to the phenotype of the cell (small, chondrocyte-like, or large notochordal). In the Capello et al. study, NCD discs that contained predominantly large notochordal cells (>99 %) were found to synthesize and distribute low molecular weight proteoglycan aggregates into the intercellular phase at a much faster (threefold) rate than did the small NP cells obtained from CD discs, allowing the aggregates to be formed farther from the cell surface. However, the CD NP-derived small chondrocyte-like cells synthesized high molecular weight proteoglycans that were rapidly assembled as high molecular weight aggregates within both the pericellular and intercellular compartments, a phenomenon considered to impose a tendency for reduced matrix remodeling and repair capacity. We observed that the NCD NP is highly cellular (predominantly physaliferous-appearing notochordal cells) with a cobweb-appearing ECM tightly held between the cells. In contrast, the CD animals contain many small clusters of cells with intense pericellular staining for aggrecan, decorin, biglycan, and fibromodulin surrounding the cells, as well as large patches of diffusely stained ECM with a distinctly fibrocartilaginous appearance.

The IVD NP of CD animals has been characterized as degenerating prematurely compared with NCD animals, and possible genetic reasons for this advanced aging and degeneration have been hypothesized [14, 30]. Furthermore, on the basis of gross pathology, histology, and MMP activity, it has been demonstrated that DDD is a common occurrence in dogs just as it is in humans, with the CD dogs experiencing these changes much earlier in life [14]. The GAG content within the NP of CD dogs is less than that found within NCD dogs, a finding consistent with the premature degeneration that occurs within this subspecies, just as are MRI classifications that are strikingly similar to those in humans, strongly supporting the role of the canine as a model of human DDD [14]. The relatively high ratio of ECM proteins in CD NP samples, fragmentation of decorin and biglycan, and sparse clusters of cells in the CD samples suggest that, akin to the degenerative human IVD NP, the CD IVD NP represents a naturally occurring degenerative phenotype in contrast to the non-degenerative NCD NP [4]. Further support for this hypothesis is that not all humans develop DDD as opposed to aging, suggesting an underlying genetic switch or series of switches that may predispose some people to DDD [31, 32] The CD canine may represent a similar genetically predetermined subset of animals that, like humans, is notochordal cell–deficient and susceptible to degenerative change [12]. A number of published studies have begun to shed some light on possible genetic susceptibility that could contribute to the development of DDD in some people (such as vitamin D receptor, collagen type XI, and others); however, this area of investigation remains to be elucidated [33–35]. The alternative hypothesis is that the CD IVD, having lost its developmentally notable notochordal cell population, has assumed a fibrocartilaginous phenotype (cartilage like cells) with much more secreted matrix, and therefore the differences we have detected reflect accelerated turnover within the more fibrocartilaginous IVD NP. This alternative hypothesis actually supports an important observation: that the fibrocartilaginous IVD NP inclusive of fragmented SLRPs and labile ECM molecules that are not tightly bound within the matrix in fact actually epitomize the degenerative NP phenotype. We strongly feel that our data dovetail with those reported by Brown et al., who demonstrated the same fragmented SLRP core proteins in degenerative human NP that we detected within the CD IVD NP [4]. Furthermore, if this fibrocartilaginous phenotype were healthy, it would not explain the fragmentation of the SLRPs or the impaired biomechanics compared with the NCD animal.

To test our hypothesis that the notochordal cell–rich IVD NP confers more optimal biomechanical properties upon the NP, in this study we measured the moment-rotation response of CD and NCD specimens during the application of a 1 Nm bending moment around the flexion and extension and 4.5 Nm around the rotational axis. The unconstrained force control testing procedure allowed the application of loads about a specific axis at the same time as minimizing the loads in the other axes of movement. The biomechanical results of the current study showed a statistically significant difference between the displacements of CD and NCD spines during flexion and extension and axial rotation movements. The flexion and extension and axial rotation parameters of the CD and NCD IVDs in our study indicate that the more degenerative and fibrocartilaginous CD IVD incurs greater displacement than does the NCD IVD, which is highly notochordal, highly hydrated, and non-degenerative. Our observations using this canine model are in agreement with the study by Iatridis et al. [5], who observed that the increase in solidlike behavior of degenerative human IVDs (compared with the more fluidlike IVD NP of youthful, non-degenerative discs) and their concomitant decreased hydrostatic pressurization reduced energy dissipation within the discs examined.

Some limitations when interpreting our present biomechanical results are noteworthy. For example, it is possible that there could be minor differences in anatomy, morphology, and biochemistry between specimens involved in this study. However, previous studies have described similar macroscopic morphological, histological, and biochemical changes in IVD degeneration in CD and NCD dogs [14, 36]. Additionally, a decrease in proteoglycan content in the NP has been considered a governing factor affecting the dynamic viscoelastic properties of the entire disc [37]. Importantly, the results of this study involved the analysis of movements with data normalized to the IVDs’ cross-sectional area, accounting for differences in IVD sizes. Therefore, despite the potential differences between CD and NCD spines and IVDs in this study, these are likely to not significantly impact our results.

## Conclusions

The CD canine NP represents a naturally occurring degenerative phenotype that shares many features of the degenerative human disc, notably fragmented SLRPs such as decorin and biglycan, the expression of which likely potentiates further ECM degradation and degeneration of the IVD. Our data suggest that the compromised biomechanical properties of the degenerative disc arise at least in part from fibrocartilaginous metaplasia of the NP secondary to fragmentation of these SLRP core proteins and the associated loss of ECM homeostasis. These observations add quantitative proteomic and biomechanical data that support the use of the dog as an optimal model with which to study human DDD and the evaluation of potential biologic therapeutics [14]. This study demonstrates that the degenerative changes that naturally occur within the CD NP make this animal a valuable animal model with which to study IVD degeneration and potential biologic therapeutics.

## Additional file

Additional file 1: Table S1. Complete data set for iTRAQ analysis of differential NP homogenate protein expression (NCD and CD animals). This table presents the accession number and names of all proteins detected within the NP homogenates of the two subspecies of canine. (PDF 151 kb)

## Abbreviations

AF: Annulus fibrosus
BSA: Bovine serum albumin
CD: Chondrodystrophic
CI: Confidence interval
COMP: Cartilage oligomeric matrix protein
DDD: Degenerative disc disease
ECM: Extracellular matrix
EDTA: Ethylenediaminetetraacetic acid
FDR: False discovery rate
GAG: Glycosaminoglycan
HAPLN1: Hyaluronan and proteoglycan link protein 1
HRP: Horseradish peroxidase
IgG: Immunoglobulin G
iTRAQ: Isobaric tags for relative and absolute quantitation
IVD: Intervertebral disc
LC: Liquid chromatography MMP, Matrix metalloproteinase
MRI: Magnetic resonance imaging
MS: Mass spectrometry
NCD: Non-chondrodystrophic
NP: Nucleus pulposus
PBS: Phosphate-buffered saline
RP: Reversed phase
SCX: Strong cation exchange
SLRP: Small leucine-rich proteoglycan
TBS: Tris-buffered saline
TGF-β: Transforming growth factor beta
VEP: Vertebral end plate
