# Supplementary material for: The biological basis of degenerative disc disease: proteomic and biomechanical analysis of the canine intervertebral disc
Source: Arthritis Res Ther. 2015 Sep 5;17(1):240. doi: 10.1186/s13075-015-0733-z (PMC4560915; doi:10.1186/s13075-015-0733-z)
Supplement: Additional file 1: Table S1. — Complete data set for iTRAQ analysis of differential NP homogenate protein expression (NCD and CD animals). This table presents the accession number and names of all proteins detected within the NP homogenates of the two subspecies of canine. (PDF 151 kb) [file 13075_2015_733_MOESM1_ESM.pdf]

Protein Ratio Feature Table		
Master N	Accession	Name
1	sp|Q28343|PGCA_CANFA	Aggrecan core protein OS=Canis familiaris GN=ACAN PE=2 SV=2
2	sp|P07589|FINC_BOVIN	Fibronectin OS=Bos taurus GN=FN1 PE=1 SV=4
3	sp|P08670|VIME_HUMAN	Vimentin OS=Homo sapiens GN=VIM PE=1 SV=4
4	sp|Q13813-2|SPTA2_HUMAN	Isoform 2 of Spectrin alpha chain, brain OS=Homo sapiens GN=SPTAN1
5	sp|P17182|ENOA_MOUSE	Alpha-enolase OS=Mus musculus GN=Eno1 PE=1 SV=3
6	sp|P08728|K1C19_BOVIN	Keratin, type I cytoskeletal 19 OS=Bos taurus GN=KRT19 PE=2 SV=1
7	sp|P25473|CLUS_CANFA	Clusterin OS=Canis familiaris GN=CLU PE=2 SV=1
8	sp|P05787|K2C8_HUMAN	Keratin, type II cytoskeletal 8 OS=Homo sapiens GN=KRT8 PE=1 SV=7
9	sp|Q4KYY3|G3P_SPECI	Glyceraldehyde-3-phosphate dehydrogenase OS=Spermophilus citellus GN=GAPDH PE=2 SV=3
10	sp|P14618-2|KPYM_HUMAN	Isoform M1 of Pyruvate kinase isozymes M1/M2 OS=Homo sapiens GN=PKM2
11	sp|O15335|CHAD_HUMAN	Chondroadherin OS=Homo sapiens GN=CHAD PE=2 SV=2
12	sp|Q62261|SPTB2_MOUSE	Spectrin beta chain, brain 1 OS=Mus musculus GN=Sptbn1 PE=1 SV=2
13	sp|Q7YQC6|HSP71_CANFA	Heat shock 70 kDa protein 1 OS=Canis familiaris GN=HSPA1 PE=2 SV=1
14	sp|Q9NZN4|EHD2_HUMAN	EH domain-containing protein 2 OS=Homo sapiens GN=EHD2 PE=1 SV=2
15	sp|Q3T0P6|PGK1_BOVIN	Phosphoglycerate kinase 1 OS=Bos taurus GN=PGK1 PE=2 SV=3
16	sp|P12111|CO6A3_HUMAN	Collagen alpha-3(VI) chain OS=Homo sapiens GN=COL6A3 PE=1 SV=4
17	sp|Q29393|PGS2_CANFA	Decorin OS=Canis familiaris GN=DCN PE=2 SV=2
18	sp|A5D7D1|ACTN4_BOVIN	Alpha-actinin-4 OS=Bos taurus GN=ACTN4 PE=2 SV=1
19	sp|P63261|ACTG_HUMAN	Actin, cytoplasmic 2 OS=Homo sapiens GN=ACTG1 PE=1 SV=1
20	sp|P02458|CO2A1_HUMAN	Collagen alpha-1(II) chain OS=Homo sapiens GN=COL2A1 PE=1 SV=3
21	sp|Q5QQ56|XYLT1_CANFA	Xylosyltransferase 1 OS=Canis familiaris GN=XYLT1 PE=2 SV=1
22	sp|Q09666|AHNK_HUMAN	Neuroblast differentiation-associated protein AHNAK OS=Homo sapiens GN=AHNAK PE=1 SV=2
23	sp|P54714|TPIS_CANFA	Triosephosphate isomerase OS=Canis familiaris GN=TPI1 PE=1 SV=3
24	sp|Q6TEQ7|ANXA2_CANFA	Annexin A2 OS=Canis familiaris GN=ANXA2 PE=1 SV=1
25	sp|A1A4K5|ENPP2_BOVIN	Ectonucleotide pyrophosphatase/phosphodiesterase family member 2 OS=Bos taurus GN=ENPP2 PE=1 SV=1
26	sp|P16015|CAH3_MOUSE	Carbonic anhydrase 3 OS=Mus musculus GN=Ca3 PE=1 SV=3
27	sp|Q9NZN3|EHD3_HUMAN	EH domain-containing protein 3 OS=Homo sapiens GN=EHD3 PE=1 SV=2
28	sp|Q6B4U9|PRDX1_MYOLU	Peroxiredoxin-1 OS=Myotis lucifugus GN=PRDX1 PE=1 SV=1
29	sp|Q9DBJ1|PGAM1_MOUSE	Phosphoglycerate mutase 1 OS=Mus musculus GN=Pgam1 PE=1 SV=3
30	sp|Q99985|SEM3C_HUMAN	Semaphorin-3C OS=Homo sapiens GN=SEMA3C PE=1 SV=2
31	sp|P35444|COMP_RAT	Cartilage oligomeric matrix protein OS=Rattus norvegicus GN=Comp PE=1 SV=1
32	sp|P30427|PLEC_RAT	Plectin OS=Rattus norvegicus GN=Plec PE=1 SV=2
33	sp|Q4R5B6|LDHB_MACFA	L-lactate dehydrogenase B chain OS=Macaca fascicularis GN=LDHB PE=2 SV=3
34	sp|P04075|ALDOA_HUMAN	Fructose-bisphosphate aldolase A OS=Homo sapiens GN=ALDOA PE=1 SV=2
35	sp|Q258K2|MYH9_CANFA	Myosin-9 OS=Canis familiaris GN=MYH9 PE=2 SV=1
36	sp|P05784|K1C18_MOUSE	Keratin, type I cytoskeletal 18 OS=Mus musculus GN=Krt18 PE=1 SV=5
37	sp|P26038|MOES_HUMAN	Moesin OS=Homo sapiens GN=MSN PE=1 SV=3
38	sp|P62936|PPIA_PIG	Peptidyl-prolyl cis-trans isomerase A OS=Sus scrofa GN=PPIA PE=1 SV=2
39	sp|P47942|DPYL2_RAT	Dihydropyrimidinase-related protein 2 OS=Rattus norvegicus GN=Dpysl2 PE=1 SV=1
40	sp|Q28275|FINC_CANFA	Fibronectin (Fragment) OS=Canis familiaris GN=FN1 PE=2 SV=2
41	sp|Q66K08|CILP1_MOUSE	Cartilage intermediate layer protein 1 OS=Mus musculus GN=Cilp PE=2 SV=1
42	sp|Q6NZI2|PTRF_HUMAN	Polymerase I and transcript release factor OS=Homo sapiens GN=PTRF PE=1 SV=1
43	sp|O75094|SLIT3_HUMAN	Slit homolog 3 protein OS=Homo sapiens GN=SLIT3 PE=2 SV=2
44	sp|Q6P9T8|TBB2C_RAT	Tubulin beta-2C chain OS=Rattus norvegicus GN=Tubb2c PE=1 SV=1
45	sp|P19858|LDHA_BOVIN	L-lactate dehydrogenase A chain OS=Bos taurus GN=LDHA PE=2 SV=2
46	sp|O75369|FLNB_HUMAN	Filamin-B OS=Homo sapiens GN=FLNB PE=1 SV=2
47	sp|P08059|G6PI_PIG	Glucose-6-phosphate isomerase OS=Sus scrofa GN=GPI PE=1 SV=3
48	sp|Q7Z7G0|TARSH_HUMAN	Target of Nesh-SH3 OS=Homo sapiens GN=ABI3BP PE=1 SV=1
49	sp|P02751|FINC_HUMAN	Fibronectin OS=Homo sapiens GN=FN1 PE=1 SV=4
50	sp|Q16851|UGPA_HUMAN	UTP--glucose-1-phosphate uridylyltransferase OS=Homo sapiens GN=UGP2 PE=1 SV=5
51	sp|Q28381|HPLN1_HORSE	Hyaluronan and proteoglycan link protein 1 OS=Equus caballus GN=HAPLN1 PE=2 SV=1
52	sp|Q71U36|TBA1A_HUMAN	Tubulin alpha-1A chain OS=Homo sapiens GN=TUBA1A PE=1 SV=1
53	sp|P10412|H14_HUMAN	Histone H1.4 OS=Homo sapiens GN=HIST1H1E PE=1 SV=2
54	sp|P18649|APOE_CANFA	Apolipoprotein E OS=Canis familiaris GN=APOE PE=1 SV=2
55	sp|Q66RN5|EF1A1_FELCA	Elongation factor 1-alpha 1 OS=Felis catus GN=EEF1A1 PE=2 SV=1
56	sp|O02678|PGS1_CANFA	Biglycan OS=Canis familiaris GN=BGN PE=2 SV=1
57	sp|Q10758|K2C8_RAT	Keratin, type II cytoskeletal 8 OS=Rattus norvegicus GN=Krt8 PE=1 SV=3
58	sp|Q71U34|HSP7C_SAGOE	Heat shock cognate 71 kDa protein OS=Saguinus oedipus GN=HSPA8 PE=2 SV=1
59	sp|P05124|KCRB_CANFA	Creatine kinase B-type OS=Canis familiaris GN=CKB PE=1 SV=1
60	sp|Q2PFV7|ACTN1_MACFA	Alpha-actinin-1 OS=Macaca fascicularis GN=ACTN1 PE=2 SV=1
61	sp|P49822|ALBU_CANFA	Serum albumin OS=Canis familiaris GN=ALB PE=1 SV=3
62	sp|P31404|VATA_BOVIN	V-type proton ATPase catalytic subunit A OS=Bos taurus GN=ATP6V1A PE=2 SV=2
63	sp|P23528|COF1_HUMAN	Cofilin-1 OS=Homo sapiens GN=CFL1 PE=1 SV=3
64	sp|P29361|1433Z_SHEEP	14-3-3 protein zeta/delta OS=Ovis aries GN=YWHAZ PE=1 SV=1
65	sp|P12109|CO6A1_HUMAN	Collagen alpha-1(VI) chain OS=Homo sapiens GN=COL6A1 PE=1 SV=3
66	sp|Q68BL8|OLM2B_HUMAN	Olfactomedin-like protein 2B OS=Homo sapiens GN=OLFML2B PE=2 SV=2
67	sp|P07900-2|HS90A_HUMAN	Isoform HSP90AA1-2 of Heat shock protein HSP 90-alpha OS=Homo sapiens GN=HSP90AA1
68	sp|P38486|LEG3_CANFA	Galectin-3 OS=Canis familiaris GN=LGALS3 PE=1 SV=3
69	sp|Q63279|K1C19_RAT	Keratin, type I cytoskeletal 19 OS=Rattus norvegicus GN=Krt19 PE=1 SV=2
70	sp|P48678|LMNA_MOUSE	Lamin-A/C OS=Mus musculus GN=Lmna PE=1 SV=2

71	sp|P98160|PGBM_HUMAN	Basement membrane-specific heparan sulfate proteoglycan core protein OS=Homo sapiens GN=HSPG2 PE=1 SV=3
72	sp|P42929|HSPB1_CANFA	Heat shock protein beta-1 OS=Canis familiaris GN=HSPB1 PE=2 SV=1
73	sp|P60526|HBB_CHRBR	Hemoglobin subunit beta OS=Chrysocyon brachyurus GN=HBB PE=1 SV=1
74	sp|P18206|VINC_HUMAN	Vinculin OS=Homo sapiens GN=VCL PE=1 SV=4
75	sp|Q3YIX4|PEBP1_CANFA	Phosphatidylethanolamine-binding protein 1 OS=Canis familiaris GN=PEBP1 PE=1 SV=1
76	sp|P47856|GFPT1_MOUSE	Glucosamine--fructose-6-phosphate aminotransferase [isomerizing] 1 OS=Mus musculus GN=Gfpt1 PE=1 SV=3
77	sp|P02510|CRYAB_BOVIN	Alpha-crystallin B chain OS=Bos taurus GN=CRYAB PE=1 SV=2
78	sp|Q9R118|HTRA1_MOUSE	Serine protease HTRA1 OS=Mus musculus GN=Htra1 PE=2 SV=1
79	sp|O77834|PRDX6_BOVIN	Peroxiredoxin-6 OS=Bos taurus GN=PRDX6 PE=1 SV=3
80	sp|P21333|FLNA_HUMAN	Filamin-A OS=Homo sapiens GN=FLNA PE=1 SV=4
81	sp|Q9EPW4|CLC3A_MOUSE	C-type lectin domain family 3 member A OS=Mus musculus GN=Clec3a PE=2 SV=1
82	sp|P51884|LUM_HUMAN	Lumican OS=Homo sapiens GN=LUM PE=1 SV=2
83	sp|Q9JM99-2|PRG4_MOUSE	Isoform B of Proteoglycan 4 OS=Mus musculus GN=Prg4
84	sp|Q32S24|COBA2_BOVIN	Collagen alpha-2(XI) chain OS=Bos taurus GN=COL11A2 PE=3 SV=1
85	sp|P60530|HBA_CANLA	Hemoglobin subunit alpha OS=Canis latrans GN=HBA PE=1 SV=2
86	sp|P81709|LYSC2_CANFA	Lysozyme C, spleen isozyme OS=Canis familiaris PE=1 SV=1
87	sp|P13608|PGCA_BOVIN	Aggrecan core protein OS=Bos taurus GN=ACAN PE=1 SV=3
88	sp|P53396|ACLY_HUMAN	ATP-citrate synthase OS=Homo sapiens GN=ACLY PE=1 SV=3
89	sp|Q3ZBZ8|STIP1_BOVIN	Stress-induced-phosphoprotein 1 OS=Bos taurus GN=STIP1 PE=2 SV=1
90	sp|P12110|CO6A2_HUMAN	Collagen alpha-2(VI) chain OS=Homo sapiens GN=COL6A2 PE=1 SV=4
91	sp|Q5R8Z3|EF2_PONAB	Elongation factor 2 OS=Pongo abelii GN=EEF2 PE=2 SV=3
92	sp|P00918|CAH2_HUMAN	Carbonic anhydrase 2 OS=Homo sapiens GN=CA2 PE=1 SV=2
93	sp|P07093|GDN_HUMAN	Glia-derived nexin OS=Homo sapiens GN=SERPINE2 PE=1 SV=1
94	sp|Q64610|ENPP2_RAT	Ectonucleotide pyrophosphatase/phosphodiesterase family member 2 OS=Rattus norvegicus GN=Enpp2 PE=1 SV=2
95	sp|Q99497|PARK7_HUMAN	Protein DJ-1 OS=Homo sapiens GN=PARK7 PE=1 SV=2
96	sp|Q6T308|GLGB_FELCA	1,4-alpha-glucan-branching enzyme OS=Felis catus GN=GBE1 PE=2 SV=1
97	sp|Q9H4F8-2|SMOC1_HUMAN	Isoform 2 of SPARC-related modular calcium-binding protein 1 OS=Homo sapiens GN=SMOC1
98	sp|P62992|RS27A_BOVIN	Ubiquitin-40S ribosomal protein S27a OS=Bos taurus GN=RPS27A PE=1 SV=2
99	sp|P00761|TRYP_PIG	Trypsin OS=Sus scrofa PE=1 SV=1
100	sp|Q9GKN8|PRELP_BOVIN	Prolargin OS=Bos taurus GN=PRELP PE=2 SV=1
101	sp|O00394|A1AT_CERAE	Alpha-1-antitrypsin (Fragment) OS=Cercopithecus aethiops GN=SERPINA1 PE=2 SV=1
102	sp|Q7YQD7|FBLN3_MACFA	EGF-containing fibulin-like extracellular matrix protein 1 OS=Macaca fascicularis GN=EFEMP1 PE=2 SV=1
103	sp|P19803|GDIR1_BOVIN	Rho GDP-dissociation inhibitor 1 OS=Bos taurus GN=ARHGDIA PE=1 SV=3
104	sp|P06396|GELS_HUMAN	Gelsolin OS=Homo sapiens GN=GSN PE=1 SV=1
105	sp|P58771-2|TPM1_MOUSE	Isoform Fibroblast of Tropomyosin alpha-1 chain OS=Mus musculus GN=Tpm1
106	sp|Q5R7K9|EDIL3_PONAB	EGF-like repeat and discoidin I-like domain-containing protein 3 OS=Pongo abelii GN=EDIL3 PE=2 SV=1
107	sp|P50609|FMOD_RAT	Fibromodulin OS=Rattus norvegicus GN=Fmod PE=2 SV=1
108	sp|Q7YRU4|MDHC_FELCA	Malate dehydrogenase, cytoplasmic OS=Felis catus GN=MDH1 PE=2 SV=3
109	sp|P11980-2|KPYM_RAT	Isoform M2 of Pyruvate kinase isozymes M1/M2 OS=Rattus norvegicus GN=Pkm2
110	sp|Q0VCX2|GRP78_BOVIN	78 kDa glucose-regulated protein OS=Bos taurus GN=HSPA5 PE=2 SV=1
111	sp|O97555|GDIA_CANFA	Rab GDP dissociation inhibitor alpha OS=Canis familiaris GN=GDI1 PE=2 SV=1
112	sp|Q92954-2|PRG4_HUMAN	Isoform B of Proteoglycan 4 OS=Homo sapiens GN=PRG4
113	sp|Q99536|VAT1_HUMAN	Synaptic vesicle membrane protein VAT-1 homolog OS=Homo sapiens GN=VAT1 PE=1 SV=2
114	sp|P62262|1433E_SHEEP	14-3-3 protein epsilon OS=Ovis aries GN=YWHAE PE=1 SV=1
115	sp|Q68FD5|CLH_MOUSE	Clathrin heavy chain 1 OS=Mus musculus GN=Cltc PE=1 SV=3
116	sp|Q5R4D4|NP1L1_PONAB	Nucleosome assembly protein 1-like 1 OS=Pongo abelii GN=NAP1L1 PE=2 SV=1
117	sp|P24821|TENA_HUMAN	Tenascin OS=Homo sapiens GN=TNC PE=1 SV=3
118	sp|P23352|KALM_HUMAN	Anosmin-1 OS=Homo sapiens GN=KAL1 PE=1 SV=3
119	sp|Q63355|MYO1C_RAT	Myosin-Ic OS=Rattus norvegicus GN=Myo1c PE=2 SV=2
120	sp|Q9XSA7|CLIC4_BOVIN	Chloride intracellular channel protein 4 OS=Bos taurus GN=CLIC4 PE=2 SV=3
121	sp|P12107-2|COBA1_HUMAN	Isoform B of Collagen alpha-1(XI) chain OS=Homo sapiens GN=COL11A1
122	sp|Q50KA8|NDKB_CANFA	Nucleoside diphosphate kinase B OS=Canis familiaris GN=NME2 PE=2 SV=1
123	sp|P79385|MFGM_PIG	Lactadherin OS=Sus scrofa GN=MFGE8 PE=1 SV=2
124	sp|P52907|CAZA1_HUMAN	F-actin-capping protein subunit alpha-1 OS=Homo sapiens GN=CAPZA1 PE=1 SV=3
125	sp|P53534|PYGB_RAT	Glycogen phosphorylase, brain form (Fragment) OS=Rattus norvegicus GN=Pygb PE=1 SV=3
126	sp|Q5R9X1|CADH2_PONAB	Cadherin-2 OS=Pongo abelii GN=CDH2 PE=2 SV=1
127	sp|Q8R1M2|H2AJ_MOUSE	Histone H2A.J OS=Mus musculus GN=H2afj PE=2 SV=1
128	sp|A3KMV5|UBA1_BOVIN	Ubiquitin-like modifier-activating enzyme 1 OS=Bos taurus GN=UBA1 PE=2 SV=1
129	sp|Q5RCP8|H2B2E_PONAB	Histone H2B type 2-E OS=Pongo abelii GN=HIST2H2BE PE=2 SV=3
130	sp|P31836|NCAM1_BOVIN	Neural cell adhesion molecule 1 OS=Bos taurus GN=NCAM1 PE=1 SV=1
131	sp|P01023|A2MG_HUMAN	Alpha-2-macroglobulin OS=Homo sapiens GN=A2M PE=1 SV=2
132	sp|P52209|6PGD_HUMAN	6-phosphogluconate dehydrogenase, decarboxylating OS=Homo sapiens GN=PGD PE=1 SV=3
133	sp|P48616|VIME_BOVIN	Vimentin OS=Bos taurus GN=VIM PE=1 SV=3
134	sp|Q8IUL8|CILP2_HUMAN	Cartilage intermediate layer protein 2 OS=Homo sapiens GN=CILP2 PE=2 SV=2
135	sp|Q9UKZ9|PCOC2_HUMAN	Procollagen C-endopeptidase enhancer 2 OS=Homo sapiens GN=PCOLCE2 PE=1 SV=1
136	sp|P00450|CERU_HUMAN	Ceruloplasmin OS=Homo sapiens GN=CP PE=1 SV=1
137	sp|P33724|CAV1_CANFA	Caveolin-1 OS=Canis familiaris GN=CAV1 PE=1 SV=1
138	sp|P51662|ANXA1_RABIT	Annexin A1 OS=Oryctolagus cuniculus GN=ANXA1 PE=1 SV=1
139	sp|Q5RCS7|H4_PONAB	Histone H4 OS=Pongo abelii PE=3 SV=1
140	sp|Q3ZBT1|TERA_BOVIN	Transitional endoplasmic reticulum ATPase OS=Bos taurus GN=VCP PE=1 SV=1
141	sp|P05786|K2C8_BOVIN	Keratin, type II cytoskeletal 8 OS=Bos taurus GN=KRT8 PE=2 SV=3
142	sp|P35445|COMP_BOVIN	Cartilage oligomeric matrix protein OS=Bos taurus GN=COMP PE=1 SV=2
143	sp|P17183|ENOG_MOUSE	Gamma-enolase OS=Mus musculus GN=Eno2 PE=1 SV=2
144	sp|O46379|LUM_RABIT	Lumican (Fragment) OS=Oryctolagus cuniculus GN=LUM PE=2 SV=1
145	sp|Q5R1X4|ALDOC_PANTR	Fructose-bisphosphate aldolase C OS=Pan troglodytes GN=ALDOC PE=2 SV=3
146	sp|Q9TS87|TAGL_BOVIN	Transgelin OS=Bos taurus GN=TAGLN PE=1 SV=4
147	sp|O62644|LECT2_BOVIN	Leukocyte cell-derived chemotaxin-2 OS=Bos taurus GN=LECT2 PE=1 SV=1
148	sp|Q7LGC8|CHST3_HUMAN	Carbohydrate sulfotransferase 3 OS=Homo sapiens GN=CHST3 PE=1 SV=3
149	sp|Q6YNX6|CALM_SHEEP	Calmodulin OS=Ovis aries GN=CALM2 PE=2 SV=3
150	sp|P53601-2|A4_MACFA	Isoform APP695 of Amyloid beta A4 protein OS=Macaca fascicularis GN=APP
151	sp|Q8HZQ5|EZRI_RABIT	Ezrin OS=Oryctolagus cuniculus GN=EZR PE=1 SV=3
152	sp|P80895|PIMT_PIG	Protein-L-isoaspartate(D-aspartate) O-methyltransferase OS=Sus scrofa GN=PCMT1 PE=1 SV=3
153	sp|Q13642-1|FHL1_HUMAN	Isoform SLIM1 of Four and a half LIM domains protein 1 OS=Homo sapiens GN=FHL1
154	sp|P54652|HSP72_HUMAN	Heat shock-related 70 kDa protein 2 OS=Homo sapiens GN=HSPA2 PE=1 SV=1
155	sp|P62815|VATB2_RAT	V-type proton ATPase subunit B, brain isoform OS=Rattus norvegicus GN=Atp6v1b2 PE=1 SV=1
156	sp|Q5IS74|TPP1_PANTR	Tripeptidyl-peptidase 1 OS=Pan troglodytes GN=TPP1 PE=2 SV=1
157	sp|Q64119|MYL6_RAT	Myosin light polypeptide 6 OS=Rattus norvegicus GN=Myl6 PE=1 SV=3
158	sp|Q710C4|SAHH_PIG	Adenosylhomocysteinase OS=Sus scrofa GN=AHCY PE=3 SV=3
159	sp|P50994|ANXA4_CANFA	Annexin A4 OS=Canis familiaris GN=ANXA4 PE=1 SV=2
160	sp|O55226|CHAD_MOUSE	Chondroadherin OS=Mus musculus GN=Chad PE=2 SV=1

161	sp|P14141|CAH3_RAT	Carbonic anhydrase 3 OS=Rattus norvegicus GN=Ca3 PE=1 SV=3
162	sp|Q8WNN6|SODC_CANFA	Superoxide dismutase [Cu-Zn] OS=Canis familiaris GN=SOD1 PE=2 SV=1
163	sp|Q13126|MTAP_HUMAN	S-methyl-5'-thioadenosine phosphorylase OS=Homo sapiens GN=MTAP PE=1 SV=2
164	sp|Q5R8S9|K1C19_PONAB	Keratin, type I cytoskeletal 19 OS=Pongo abelii GN=KRT19 PE=2 SV=1
165	sp|P11276|FINC_MOUSE	Fibronectin OS=Mus musculus GN=Fn1 PE=1 SV=3
166	sp|P05783|K1C18_HUMAN	Keratin, type I cytoskeletal 18 OS=Homo sapiens GN=KRT18 PE=1 SV=2
167	sp|Q2PFZ3|PRDX2_MACFA	Peroxiredoxin-2 OS=Macaca fascicularis GN=PRDX2 PE=2 SV=3
168	sp|Q5R8R1|ARP3_PONAB	Actin-related protein 3 OS=Pongo abelii GN=ACTR3 PE=2 SV=3
169	sp|P56480|ATPB_MOUSE	ATP synthase subunit beta, mitochondrial OS=Mus musculus GN=Atp5b PE=1 SV=2
170	sp|Q8HZQ0|RNAS4_PANTR	Ribonuclease 4 OS=Pan troglodytes GN=RNASE4 PE=3 SV=1
171	sp|Q5REN2|PSA1_PONAB	Proteasome subunit alpha type-1 OS=Pongo abelii GN=PSMA1 PE=2 SV=1
172	sp|Q28008|CLC3A_BOVIN	C-type lectin domain family 3 member A OS=Bos taurus GN=CLEC3A PE=2 SV=1
173	sp|Q9H299|SH3L3_HUMAN	SH3 domain-binding glutamic acid-rich-like protein 3 OS=Homo sapiens GN=SH3BGRL3 PE=1 SV=1
174	sp|O76041|NEBL_HUMAN	Nebulette OS=Homo sapiens GN=NEBL PE=2 SV=1
175	sp|Q9Y490|TLN1_HUMAN	Talin-1 OS=Homo sapiens GN=TLN1 PE=1 SV=3
176	sp|Q5R4C1|TKT_PONAB	Transketolase OS=Pongo abelii GN=TKT PE=2 SV=1
177	sp|A1A4J1|K6PL_BOVIN	6-phosphofructokinase, liver type OS=Bos taurus GN=PFKL PE=2 SV=1
178	sp|A5D7R1|TR11B_BOVIN	Tumor necrosis factor receptor superfamily member 11B OS=Bos taurus GN=TNFRSF11B PE=2 SV=1
179	sp|P13697|MAOX_RAT	NADP-dependent malic enzyme OS=Rattus norvegicus GN=Me1 PE=1 SV=2
180	sp|P02459|CO2A1_BOVIN	Collagen alpha-1(II) chain (Fragments) OS=Bos taurus GN=COL2A1 PE=1 SV=3
181	sp|P13942|COBA2_HUMAN	Collagen alpha-2(XI) chain OS=Homo sapiens GN=COL11A2 PE=1 SV=4
182	sp|P80928|MIF_PIG	Macrophage migration inhibitory factor OS=Sus scrofa GN=MIF PE=1 SV=3
183	sp|Q4R5F2|LGUL_MACFA	Lactoylglutathione lyase OS=Macaca fascicularis GN=GLO1 PE=2 SV=3
184	sp|P08729|K2C7_HUMAN	Keratin, type II cytoskeletal 7 OS=Homo sapiens GN=KRT7 PE=1 SV=4
185	sp|Q2TBQ8|6PGL_BOVIN	6-phosphogluconolactonase OS=Bos taurus GN=PGLS PE=2 SV=1
186	sp|Q6YHK3|CD109_HUMAN	CD109 antigen OS=Homo sapiens GN=CD109 PE=1 SV=2
187	sp|Q9EQP5|PRELP_RAT	Prolargin OS=Rattus norvegicus GN=Prelp PE=2 SV=1
188	sp|Q6RUV5|RAC1_RAT	Ras-related C3 botulinum toxin substrate 1 OS=Rattus norvegicus GN=Rac1 PE=1 SV=1
189	sp|O95340|PAPS2_HUMAN	Bifunctional 3'-phosphoadenosine 5'-phosphosulfate synthase 2 OS=Homo sapiens GN=PAPSS2 PE=1 SV=2
190	sp|P20112|SPRC_PIG	SPARC OS=Sus scrofa GN=SPARC PE=1 SV=2
191	sp|Q91VJ2|PRDBP_MOUSE	Protein kinase C delta-binding protein OS=Mus musculus GN=Prkcdbp PE=1 SV=1
192	sp|Q7JJU6|TBB5_PANTR	Tubulin beta chain OS=Pan troglodytes GN=TUBB PE=3 SV=1
193	sp|P20618|PSB1_HUMAN	Proteasome subunit beta type-1 OS=Homo sapiens GN=PSMB1 PE=1 SV=2
194	sp|Q7SIB7|PGK1_PIG	Phosphoglycerate kinase 1 OS=Sus scrofa GN=PGK1 PE=1 SV=3
195	sp|Q5R1W0|ANXA5_PANTR	Annexin A5 OS=Pan troglodytes GN=ANXA5 PE=2 SV=3
196	sp|P06733|ENOA_HUMAN	Alpha-enolase OS=Homo sapiens GN=ENO1 PE=1 SV=2
197	sp|P07451|CAH3_HUMAN	Carbonic anhydrase 3 OS=Homo sapiens GN=CA3 PE=1 SV=3
198	sp|Q61792|LASP1_MOUSE	LIM and SH3 domain protein 1 OS=Mus musculus GN=Lasp1 PE=1 SV=1
199	sp|P05114|HMGN1_HUMAN	Non-histone chromosomal protein HMG-14 OS=Homo sapiens GN=HMGN1 PE=1 SV=3
200	sp|Q5RC20|1433G_PONAB	14-3-3 protein gamma OS=Pongo abelii GN=YWHAG PE=2 SV=3
201	sp|Q66H80|COPD_RAT	Coatomer subunit delta OS=Rattus norvegicus GN=Arcn1 PE=2 SV=1
202	sp|Q11011|PSA_MOUSE	Puromycin-sensitive aminopeptidase OS=Mus musculus GN=Npepps PE=1 SV=2
203	sp|P11679|K2C8_MOUSE	Keratin, type II cytoskeletal 8 OS=Mus musculus GN=Krt8 PE=1 SV=4
204	sp|P02639|S10A1_BOVIN	Protein S100-A1 OS=Bos taurus GN=S100A1 PE=1 SV=2
205	sp|Q29443|TRFE_BOVIN	Serotransferrin OS=Bos taurus GN=TF PE=2 SV=1
206	sp|Q8VHX6|FLNC_MOUSE	Filamin-C OS=Mus musculus GN=Flnc PE=1 SV=3
207	sp|Q76M96|CCD80_HUMAN	Coiled-coil domain-containing protein 80 OS=Homo sapiens GN=CCDC80 PE=1 SV=1
208	sp|Q9P0L0|VAPA_HUMAN	Vesicle-associated membrane protein-associated protein A OS=Homo sapiens GN=VAPA PE=1 SV=3
209	sp|P13355|GTR1_RABIT	Solute carrier family 2, facilitated glucose transporter member 1 OS=Oryctolagus cuniculus GN=SLC2A1 PE=2 SV=1
210	sp|Q62159|RHOC_MOUSE	Rho-related GTP-binding protein RhoC OS=Mus musculus GN=Rhoc PE=1 SV=2
211	sp|O08529|CAN2_MOUSE	Calpain-2 catalytic subunit OS=Mus musculus GN=Capn2 PE=2 SV=4
212	sp|Q9TQX6|ACBP_CANFA	Acyl-CoA-binding protein OS=Canis familiaris GN=DBI PE=1 SV=2
213	sp|P40227|TCPZ_HUMAN	T-complex protein 1 subunit zeta OS=Homo sapiens GN=CCT6A PE=1 SV=3
214	sp|Q5RBU8|ROA2_PONAB	Heterogeneous nuclear ribonucleoproteins A2/B1 OS=Pongo abelii GN=HNRNPA2B1 PE=2 SV=1
215	sp|Q8BG05|ROA3_MOUSE	Heterogeneous nuclear ribonucleoprotein A3 OS=Mus musculus GN=Hnrnpa3 PE=1 SV=1
216	sp|Q5RER0|ITIH5_PONAB	Inter-alpha-trypsin inhibitor heavy chain H5 OS=Pongo abelii GN=ITIH5 PE=2 SV=1
217	sp|Q8R491|EHD3_RAT	EH domain-containing protein 3 OS=Rattus norvegicus GN=Ehd3 PE=1 SV=2
218	sp|Q00612|G6PD1_MOUSE	Glucose-6-phosphate 1-dehydrogenase X OS=Mus musculus GN=G6pdx PE=1 SV=3
219	sp|P79136|CAPZB_BOVIN	F-actin-capping protein subunit beta OS=Bos taurus GN=CAPZB PE=1 SV=1
220	sp|Q76MZ3|2AAA_MOUSE	Serine/threonine-protein phosphatase 2A 65 kDa regulatory subunit A alpha isoform OS=Mus musculus GN=Ppp2r1a PE=1 SV=3
221	sp|P06151|LDHA_MOUSE	L-lactate dehydrogenase A chain OS=Mus musculus GN=Ldha PE=1 SV=3
222	sp|Q9GKX8|HS90B_HORSE	Heat shock protein HSP 90-beta OS=Equus caballus GN=HSP90AB1 PE=2 SV=3
223	sp|Q6SQH4|S10AA_RABIT	Protein S100-A10 OS=Oryctolagus cuniculus GN=S100a10 PE=3 SV=3
224	sp|Q76I81|RS12_BOVIN	40S ribosomal protein S12 OS=Bos taurus GN=RPS12 PE=2 SV=1
225	sp|A7MB70|SEM3C_BOVIN	Semaphorin-3C OS=Bos taurus GN=SEMA3C PE=2 SV=1
226	sp|Q9Z1N5|UAP56_MOUSE	Spliceosome RNA helicase Bat1 OS=Mus musculus GN=Bat1 PE=1 SV=1
227	sp|P82460|THIO_PIG	Thioredoxin OS=Sus scrofa GN=TXN PE=1 SV=3
228	sp|Q92743|HTRA1_HUMAN	Serine protease HTRA1 OS=Homo sapiens GN=HTRA1 PE=1 SV=1
229	sp|P04937-2|FINC_RAT	Isoform FNIII-13-less of Fibronectin OS=Rattus norvegicus GN=Fn1
230	sp|P09488|GSTM1_HUMAN	Glutathione S-transferase Mu 1 OS=Homo sapiens GN=GSTM1 PE=1 SV=3
231	sp|P07737|PROF1_HUMAN	Profilin-1 OS=Homo sapiens GN=PFN1 PE=1 SV=2
232	sp|Q4R502|IDHP_MACFA	Isocitrate dehydrogenase [NADP], mitochondrial OS=Macaca fascicularis GN=IDH2 PE=2 SV=1
233	sp|Q27972|CHAD_BOVIN	Chondroadherin OS=Bos taurus GN=CHAD PE=1 SV=1
234	sp|Q5R5F0|LDHA_PONAB	L-lactate dehydrogenase A chain OS=Pongo abelii GN=LDHA PE=2 SV=3
235	sp|P19001|K1C19_MOUSE	Keratin, type I cytoskeletal 19 OS=Mus musculus GN=Krt19 PE=2 SV=1
236	sp|P16403|H12_HUMAN	Histone H1.2 OS=Homo sapiens GN=HIST1H1C PE=1 SV=2
237	sp|P45591|COF2_MOUSE	Cofilin-2 OS=Mus musculus GN=Cfl2 PE=1 SV=1
238	sp|Q8CIB5|FERM2_MOUSE	Fermitin family homolog 2 OS=Mus musculus GN=Fermt2 PE=1 SV=1
239	sp|Q9Z2K9|IDHC_MICME	Isocitrate dehydrogenase [NADP] cytoplasmic OS=Microtus mexicanus GN=IDH1 PE=2 SV=1
240	sp|Q5E9E6|RL10A_BOVIN	60S ribosomal protein L10a OS=Bos taurus GN=RPL10A PE=2 SV=3
241	sp|P12830|CADH1_HUMAN	Cadherin-1 OS=Homo sapiens GN=CDH1 PE=1 SV=3
242	sp|P28075|PSB5_RAT	Proteasome subunit beta type-5 OS=Rattus norvegicus GN=Psmb5 PE=1 SV=3
243	sp|Q8CFN2|CDC42_RAT	Cell division control protein 42 homolog OS=Rattus norvegicus GN=Cdc42 PE=1 SV=2
244	sp|P08294|SODE_HUMAN	Extracellular superoxide dismutase [Cu-Zn] OS=Homo sapiens GN=SOD3 PE=1 SV=2
245	sp|Q3ZBD7|G6PI_BOVIN	Glucose-6-phosphate isomerase OS=Bos taurus GN=GPI PE=2 SV=3
246	sp|Q148J6|ARPC4_BOVIN	Actin-related protein 2/3 complex subunit 4 OS=Bos taurus GN=ARPC4 PE=1 SV=3
247	sp|O00300|TR11B_HUMAN	Tumor necrosis factor receptor superfamily member 11B OS=Homo sapiens GN=TNFRSF11B PE=1 SV=2
248	sp|P50503|F10A1_RAT	Hsc70-interacting protein OS=Rattus norvegicus GN=St13 PE=1 SV=1
249	sp|Q5R5I4|UB2L3_PONAB	Ubiquitin-conjugating enzyme E2 L3 OS=Pongo abelii GN=UBE2L3 PE=2 SV=1
250	sp|Q9D0M5|DYL2_MOUSE	Dynein light chain 2, cytoplasmic OS=Mus musculus GN=Dynll2 PE=1 SV=1
251	sp|P21741|MK_HUMAN	Midkine OS=Homo sapiens GN=MDK PE=1 SV=1

252	sp|P50518|VATE1_MOUSE	V-type proton ATPase subunit E 1 OS=Mus musculus GN=Atp6v1e1 PE=1 SV=2
253	sp|Q9TTY1|TIMP2_CANFA	Metalloproteinase inhibitor 2 OS=Canis familiaris GN=TIMP2 PE=2 SV=2
254	sp|Q71LE2|H33_PIG	Histone H3.3 OS=Sus scrofa GN=H3F3A PE=2 SV=3
255	sp|P55918|MFAP4_BOVIN	Microfibril-associated glycoprotein 4 OS=Bos taurus GN=MFAP4 PE=1 SV=2
256	sp|Q5RKI1|IF4A2_RAT	Eukaryotic initiation factor 4A-II OS=Rattus norvegicus GN=Eif4a2 PE=1 SV=1
257	sp|Q86Y38|XYLT1_HUMAN	Xylosyltransferase 1 OS=Homo sapiens GN=XYLT1 PE=1 SV=1
258	sp|Q15149-4|PLEC_HUMAN	Isoform Plectin-11 of Plectin OS=Homo sapiens GN=PLEC
259	sp|Q6EWQ7|IF5A1_BOVIN	Eukaryotic translation initiation factor 5A-1 OS=Bos taurus GN=EIF5A PE=2 SV=3
260	sp|Q9TV56|S10A4_CANFA	Protein S100-A4 OS=Canis familiaris GN=S100A4 PE=3 SV=1
261	sp|Q29116|TENA_PIG	Tenascin OS=Sus scrofa GN=TNC PE=1 SV=1
262	sp|Q865V6|CAPG_BOVIN	Macrophage-capping protein OS=Bos taurus GN=CAPG PE=2 SV=1
263	sp|O75083|WDR1_HUMAN	WD repeat-containing protein 1 OS=Homo sapiens GN=WDR1 PE=1 SV=4
264	sp|Q2EN75|S10A6_PIG	Protein S100-A6 OS=Sus scrofa GN=S100A6 PE=3 SV=1
265	sp|Q60847|COCA1_MOUSE	Collagen alpha-1(XII) chain OS=Mus musculus GN=Col12a1 PE=2 SV=3
266	sp|Q6UVK1|CSPG4_HUMAN	Chondroitin sulfate proteoglycan 4 OS=Homo sapiens GN=CSPG4 PE=1 SV=2
267	sp|O35987|NSF1C_RAT	NSFL1 cofactor p47 OS=Rattus norvegicus GN=Nsfl1c PE=1 SV=1
268	sp|Q5R556|RAN_PONAB	GTP-binding nuclear protein Ran OS=Pongo abelii GN=RAN PE=2 SV=3
269	sp|P31324|KAP3_MOUSE	cAMP-dependent protein kinase type II-beta regulatory subunit OS=Mus musculus GN=Prkar2b PE=1 SV=3
270	sp|Q9H361|PABP3_HUMAN	Polyadenylate-binding protein 3 OS=Homo sapiens GN=PABPC3 PE=1 SV=2
271	sp|O11780|BGH3_PIG	Transforming growth factor-beta-induced protein ig-h3 OS=Sus scrofa GN=TGFBI PE=1 SV=1
272	sp|Q07954|LRP1_HUMAN	Prolow-density lipoprotein receptor-related protein 1 OS=Homo sapiens GN=LRP1 PE=1 SV=1
273	sp|Q96JB1|DYH8_HUMAN	Dynein heavy chain 8, axonemal OS=Homo sapiens GN=DNAH8 PE=1 SV=2
274	sp|Q3ZC84|CNDP2_BOVIN	Cytosolic non-specific dipeptidase OS=Bos taurus GN=CNDP2 PE=2 SV=1
275	sp|P51635|AK1A1_RAT	Alcohol dehydrogenase [NADP+] OS=Rattus norvegicus GN=Akr1a1 PE=1 SV=2
276	sp|P97927|LAMA4_MOUSE	Laminin subunit alpha-4 OS=Mus musculus GN=Lama4 PE=1 SV=2
277	sp|Q13822-2|ENPP2_HUMAN	Isoform Alpha of Ectonucleotide pyrophosphatase/phosphodiesterase family member 2 OS=Homo sapiens GN=ENPP2
278	sp|Q03001-9|BPA1_HUMAN	Isoform EB of Bullous pemphigoid antigen 1 OS=Homo sapiens GN=DST
279	sp|P11586|C1TC_HUMAN	C-1-tetrahydrofolate synthase, cytoplasmic OS=Homo sapiens GN=MTHFD1 PE=1 SV=3
280	sp|P14866|HNRPL_HUMAN	Heterogeneous nuclear ribonucleoprotein L OS=Homo sapiens GN=HNRNPL PE=1 SV=2
281	sp|Q9NRN5|OLFL3_HUMAN	Olfactomedin-like protein 3 OS=Homo sapiens GN=OLFML3 PE=2 SV=1
282	sp|Q4V8H8|EHD2_RAT	EH domain-containing protein 2 OS=Rattus norvegicus GN=Ehd2 PE=1 SV=1
283	sp|Q7TMY8|HUWE1_MOUSE	E3 ubiquitin-protein ligase HUWE1 OS=Mus musculus GN=Huwe1 PE=1 SV=4
284	sp|P00570|KAD1_BOVIN	Adenylate kinase isoenzyme 1 OS=Bos taurus GN=AK1 PE=1 SV=2
285	sp|Q6P502|TCPG_RAT	T-complex protein 1 subunit gamma OS=Rattus norvegicus GN=Cct3 PE=1 SV=1
286	sp|P00813|ADA_HUMAN	Adenosine deaminase OS=Homo sapiens GN=ADA PE=1 SV=3
287	sp|Q3T035|ARPC3_BOVIN	Actin-related protein 2/3 complex subunit 3 OS=Bos taurus GN=ARPC3 PE=1 SV=3
288	sp|P46940|IQGA1_HUMAN	Ras GTPase-activating-like protein IQGAP1 OS=Homo sapiens GN=IQGAP1 PE=1 SV=1
289	sp|Q9TSZ6|DAG1_CANFA	Dystroglycan OS=Canis familiaris GN=DAG1 PE=3 SV=1

290	sp|Q5RBG1|RAB5B_PONAB	Ras-related protein Rab-5B OS=Pongo abelii GN=RAB5B PE=2 SV=1
291	sp|P70349|HINT1_MOUSE	Histidine triad nucleotide-binding protein 1 OS=Mus musculus GN=Hint1 PE=1 SV=3
292	sp|Q08DP0|PGM1_BOVIN	Phosphoglucomutase-1 OS=Bos taurus GN=PGM1 PE=2 SV=1
293	sp|Q9TUL9|TIMP3_HORSE	Metalloproteinase inhibitor 3 OS=Equus caballus GN=TIMP3 PE=2 SV=1
294	sp|P49747|COMP_HUMAN	Cartilage oligomeric matrix protein OS=Homo sapiens GN=COMP PE=1 SV=2
295	sp|Q5VYK3|ECM29_HUMAN	Proteasome-associated protein ECM29 homolog OS=Homo sapiens GN=ECM29 PE=1 SV=2
296	sp|Q63210|GNA12_RAT	Guanine nucleotide-binding protein subunit alpha-12 OS=Rattus norvegicus GN=Gna12 PE=2 SV=3
297	sp|P15170|ERF3A_HUMAN	Eukaryotic peptide chain release factor GTP-binding subunit ERF3A OS=Homo sapiens GN=GSPT1 PE=1 SV=1
298	sp|P52174|NDKA1_BOVIN	Nucleoside diphosphate kinase A 1 OS=Bos taurus GN=NME1-1 PE=1 SV=3
299	sp|Q4VIT4|PDIA3_CERAE	Protein disulfide-isomerase A3 OS=Cercopithecus aethiops GN=PDIA3 PE=2 SV=1
300	sp|Q6AY84|SCRN1_RAT	Secernin-1 OS=Rattus norvegicus GN=Scrn1 PE=1 SV=1
301	sp|Q5E984|TCTP_BOVIN	Translationally-controlled tumor protein OS=Bos taurus GN=TPT1 PE=2 SV=1
302	sp|P15311|EZRI_HUMAN	Ezrin OS=Homo sapiens GN=EZR PE=1 SV=4
303	sp|Q9JHW0|PSB7_RAT	Proteasome subunit beta type-7 OS=Rattus norvegicus GN=Psmb7 PE=1 SV=1
304	sp|Q9UL16|CCD19_HUMAN	Coiled-coil domain-containing protein 19, mitochondrial OS=Homo sapiens GN=CCDC19 PE=1 SV=2
305	sp|Q62920|PDLI5_RAT	PDZ and LIM domain protein 5 OS=Rattus norvegicus GN=Pdlim5 PE=1 SV=2
306	sp|P30044|PRDX5_HUMAN	Peroxiredoxin-5, mitochondrial OS=Homo sapiens GN=PRDX5 PE=1 SV=3
307	sp|Q9HBL0|TENS1_HUMAN	Tensin-1 OS=Homo sapiens GN=TNS1 PE=1 SV=2
308	sp|Q02809|PLOD1_HUMAN	Procollagen-lysine,2-oxoglutarate 5-dioxygenase 1 OS=Homo sapiens GN=PLOD1 PE=1 SV=2
309	sp|P63322|RALA_RAT	Ras-related protein Ral-A OS=Rattus norvegicus GN=Rala PE=1 SV=1
310	sp|Q5RB59|PUR6_PONAB	Multifunctional protein ADE2 OS=Pongo abelii GN=PAICS PE=2 SV=3
311	sp|Q86V81|THOC4_HUMAN	THO complex subunit 4 OS=Homo sapiens GN=THOC4 PE=1 SV=3
312	sp|Q9CPW4|ARPC5_MOUSE	Actin-related protein 2/3 complex subunit 5 OS=Mus musculus GN=Arpc5 PE=2 SV=3
313	sp|Q8BIZ0|PCD20_MOUSE	Protocadherin-20 OS=Mus musculus GN=Pcdh20 PE=2 SV=2
314	sp|P20072|ANXA7_BOVIN	Annexin A7 OS=Bos taurus GN=ANXA7 PE=1 SV=2
315	sp|Q9H223|EHD4_HUMAN	EH domain-containing protein 4 OS=Homo sapiens GN=EHD4 PE=1 SV=1
316	sp|Q8WNW3|PLAK_PIG	Junction plakoglobin OS=Sus scrofa GN=Jup PE=2 SV=1
317	sp|Q9JK81|MYG1_MOUSE	UPF0160 protein MYG1, mitochondrial OS=Mus musculus GN=Myg1 PE=2 SV=1
318	sp|P17980|PRS6A_HUMAN	26S protease regulatory subunit 6A OS=Homo sapiens GN=PSMC3 PE=1 SV=3
319	sp|Q5EAC6|CDC37_BOVIN	Hsp90 co-chaperone Cdc37 OS=Bos taurus GN=CDC37 PE=2 SV=1
320	sp|Q9CVB6|ARPC2_MOUSE	Actin-related protein 2/3 complex subunit 2 OS=Mus musculus GN=Arpc2 PE=1 SV=3
321	sp|P50995|ANX11_HUMAN	Annexin A11 OS=Homo sapiens GN=ANXA11 PE=1 SV=1
322	sp|Q95204|CY561_SHEEP	Cytochrome b561 OS=Ovis aries GN=CYB561 PE=2 SV=1
323	sp|P79342|S10AD_BOVIN	Protein S100-A13 OS=Bos taurus GN=S100A13 PE=3 SV=2
324	sp|Q5NVT2|ZN770_PONAB	Zinc finger protein 770 (Fragment) OS=Pongo abelii GN=ZNF770 PE=2 SV=1
325	sp|Q4PLJ0|NEDD8_RABIT	NEDD8 OS=Oryctolagus cuniculus GN=NEDD8 PE=3 SV=1
326	sp|Q9Z0N1|IF2G_MOUSE	Eukaryotic translation initiation factor 2 subunit 3, X-linked OS=Mus musculus GN=Eif2s3x PE=1 SV=2
327	sp|Q99969|RARR2_HUMAN	Retinoic acid receptor responder protein 2 OS=Homo sapiens GN=RARRES2 PE=1 SV=1
328	sp|Q6YKA4|HMGB1_CANFA	High mobility group protein B1 OS=Canis familiaris GN=HMGB1 PE=2 SV=3
329	sp|Q9N0Y0|VAMP2_MACMU	Vesicle-associated membrane protein 2 OS=Macaca mulatta GN=VAMP2 PE=3 SV=3
330	sp|Q16881|TRXR1_HUMAN	Thioredoxin reductase 1, cytoplasmic OS=Homo sapiens GN=TXNRD1 PE=1 SV=3
331	sp|Q3ZC42|ADHX_BOVIN	Alcohol dehydrogenase class-3 OS=Bos taurus GN=ADH5 PE=2 SV=1
332	sp|P33097|AATC_BOVIN	Aspartate aminotransferase, cytoplasmic OS=Bos taurus GN=GOT1 PE=1 SV=3
333	RRRRRsp|A6QPA3|BTBDJ_BOVIN	REVERSED BTB/POZ domain-containing protein 19 OS=Bos taurus GN=BTBD19 PE=2 SV=1
334	sp|P27816|MAP4_HUMAN	Microtubule-associated protein 4 OS=Homo sapiens GN=MAP4 PE=1 SV=3
335	sp|Q5R5U1|RAB10_PONAB	Ras-related protein Rab-10 OS=Pongo abelii GN=RAB10 PE=2 SV=1
336	sp|Q68FQ0|TCPE_RAT	T-complex protein 1 subunit epsilon OS=Rattus norvegicus GN=Cct5 PE=1 SV=1
337	sp|Q5RAE1|CPNE3_PONAB	Copine-3 OS=Pongo abelii GN=CPNE3 PE=2 SV=1
338	RRRRRsp|Q9D700|DUS26_MOUSE	REVERSED Dual specificity protein phosphatase 26 OS=Mus musculus GN=Dusp26 PE=2 SV=2
339	sp|O75339|CILP1_HUMAN	Cartilage intermediate layer protein 1 OS=Homo sapiens GN=CILP PE=1 SV=3
340	sp|Q9UBG0|MRC2_HUMAN	C-type mannose receptor 2 OS=Homo sapiens GN=MRC2 PE=1 SV=1
341	sp|O60763|USO1_HUMAN	General vesicular transport factor p115 OS=Homo sapiens GN=USO1 PE=1 SV=2
342	sp|P16086|SPTA2_RAT	Spectrin alpha chain, brain OS=Rattus norvegicus GN=Sptan1 PE=1 SV=2
343	sp|Q5BJY9|K1C18_RAT	Keratin, type I cytoskeletal 18 OS=Rattus norvegicus GN=Krt18 PE=1 SV=3
344	sp|P62909|RS3_RAT	40S ribosomal protein S3 OS=Rattus norvegicus GN=Rps3 PE=2 SV=1
345	sp|P80313|TCPH_MOUSE	T-complex protein 1 subunit eta OS=Mus musculus GN=Cct7 PE=1 SV=1
346	sp|Q95117|SFRP3_BOVIN	Secreted frizzled-related protein 3 OS=Bos taurus GN=FRZB PE=1 SV=1
347	sp|Q641Y8|DDX1_RAT	ATP-dependent RNA helicase DDX1 OS=Rattus norvegicus GN=Ddx1 PE=2 SV=1
348	RRRRRsp|Q9H2S5|RNF39_HUMAN	REVERSED RING finger protein 39 OS=Homo sapiens GN=RNF39 PE=1 SV=2
349	sp|Q9R1P1|PSB3_MOUSE	Proteasome subunit beta type-3 OS=Mus musculus GN=Psmb3 PE=1 SV=1
350	sp|Q3SZF2|ARF4_BOVIN	ADP-ribosylation factor 4 OS=Bos taurus GN=ARF4 PE=2 SV=3
351	sp|P05064|ALDOA_MOUSE	Fructose-bisphosphate aldolase A OS=Mus musculus GN=Aldoa PE=1 SV=2
352	sp|Q04857|CO6A1_MOUSE	Collagen alpha-1(VI) chain OS=Mus musculus GN=Col6a1 PE=2 SV=1
353	sp|Q4R572|1433B_MACFA	14-3-3 protein beta/alpha OS=Macaca fascicularis GN=YWHAB PE=2 SV=3
354	sp|Q68FP1|GELS_RAT	Gelsolin OS=Rattus norvegicus GN=Gsn PE=1 SV=1
355	sp|Q8TER5-3|SOLO_HUMAN	Isoform 3 of Protein SOLO OS=Homo sapiens GN=SOLO
356	sp|P35221-2|CTNA1_HUMAN	Isoform 2 of Catenin alpha-1 OS=Homo sapiens GN=CTNNA1
357	sp|Q10994|CYTB_SHEEP	Cystatin-B OS=Ovis aries GN=CSTB PE=1 SV=1
358	sp|O43242|PSMD3_HUMAN	26S proteasome non-ATPase regulatory subunit 3 OS=Homo sapiens GN=PSMD3 PE=1 SV=2
359	sp|Q01634|IDUA_CANFA	Alpha-L-iduronidase OS=Canis familiaris GN=IDUA PE=1 SV=1
360	sp|Q60692|PSB6_MOUSE	Proteasome subunit beta type-6 OS=Mus musculus GN=Psmb6 PE=1 SV=3
361	sp|P78417|GSTO1_HUMAN	Glutathione S-transferase omega-1 OS=Homo sapiens GN=GSTO1 PE=1 SV=2
362	sp|P47959|HPRT_MERUN	Hypoxanthine-guanine phosphoribosyltransferase OS=Meriones unguiculatus GN=HPRT1 PE=2 SV=2
363	sp|Q5T7N8|F27D1_HUMAN	Protein FAM27D1 OS=Homo sapiens GN=FAM27D1 PE=3 SV=2
364	sp|Q9XSJ4|ENOA_BOVIN	Alpha-enolase OS=Bos taurus GN=ENO1 PE=1 SV=4
365	sp|Q9H4M9|EHD1_HUMAN	EH domain-containing protein 1 OS=Homo sapiens GN=EHD1 PE=1 SV=2
366	sp|P07450|CAH3_HORSE	Carbonic anhydrase 3 OS=Equus caballus GN=CA3 PE=1 SV=2
367	sp|Q63610|TPM3_RAT	Tropomyosin alpha-3 chain OS=Rattus norvegicus GN=Tpm3 PE=1 SV=2
368	sp|Q9NQS7-2|INCE_HUMAN	Isoform 2 of Inner centromere protein OS=Homo sapiens GN=INCENP
369	sp|O70435|PSA3_MOUSE	Proteasome subunit alpha type-3 OS=Mus musculus GN=Psma3 PE=1 SV=3
370	sp|O70492|SNX3_MOUSE	Sorting nexin-3 OS=Mus musculus GN=Snx3 PE=2 SV=3
371	sp|Q9WVB4|SLIT3_MOUSE	Slit homolog 3 protein OS=Mus musculus GN=Slit3 PE=2 SV=1
372	sp|O43252|PAPS1_HUMAN	Bifunctional 3'-phosphoadenosine 5'-phosphosulfate synthase 1 OS=Homo sapiens GN=PAPSS1 PE=1 SV=2
373	sp|Q4R4I6|CAP1_MACFA	Adenylyl cyclase-associated protein 1 OS=Macaca fascicularis GN=CAP1 PE=2 SV=3
374	sp|P47199|QOR_MOUSE	Quinone oxidoreductase OS=Mus musculus GN=Cryz PE=2 SV=1
375	sp|Q61481|PDE1A_MOUSE	Calcium/calmodulin-dependent 3',5'-cyclic nucleotide phosphodiesterase 1A OS=Mus musculus GN=Pde1a PE=2 SV=1
376	sp|Q9Z204|HNRPC_MOUSE	Heterogeneous nuclear ribonucleoproteins C1/C2 OS=Mus musculus GN=Hnrnpc PE=1 SV=1
377	sp|Q6AY09|HNRH2_RAT	Heterogeneous nuclear ribonucleoprotein H2 OS=Rattus norvegicus GN=Hnrnph2 PE=1 SV=1
